# Conservatively transmitted alleles of key agronomic genes provide insights into the genetic basis of founder parents in bread wheat (*Triticum aestivum* L.)

**DOI:** 10.1186/s12870-023-04098-x

**Published:** 2023-02-18

**Authors:** Chang Li, Lei Zhuang, Tian Li, Jian Hou, Hongxia Liu, Chao Jian, Huifang Li, Jing Zhao, Yunchuan Liu, Wei Xi, Pingan Hao, Shujuan Liu, Xuemei Si, Xiaolu Wang, Xueyong Zhang, Chenyang Hao

**Affiliations:** Key Laboratory of Crop Gene Resources and Germplasm Enhancement, Ministry of Agriculture and Rural Affairs/The National Key Facility for Crop Gene Resources and Genetic Improvement/Institute of Crop Sciences, Chinese Academy of Agricultural Sciences, Beijing, 100081 China

**Keywords:** Conservative transmission allele, Agronomic gene, KASP marker, Founder parent, Common wheat

## Abstract

**Background:**

Founder parents play extremely important roles in wheat breeding. Studies into the genetic basis of founder parents and the transmission rules of favorable alleles are of great significance in improving agronomically important traits in wheat.

**Results:**

Here, a total of 366 founder parents, widely grown cultivars, and derivatives of four representative founder parents were genotyped based on efficient kompetitive allele-specific PCR (KASP) markers in 87 agronomically important genes controlling yield, quality, adaptability, and stress resistance. Genetic composition analysis of founder parents and widely grown cultivars showed a consistently high frequency of favorable alleles for yield-related genes. This analysis further showed that other alleles favorable for resistance, strong gluten, dwarf size, and early heading date were also subject to selective pressure over time. By comparing the transmission of alleles from four representative founder parents to their derivatives during different breeding periods, it was found that the genetic composition of the representative founder parents was optimized as breeding progressed over time, with the number and types of favorable alleles carried gradually increasing and becoming enriched. There are still a large number of favorable alleles in wheat founder parents that have not been fully utilized in breeding selection. Eighty-seven agronomically important genes were used to construct an enrichment map that shows favorable alleles of four founder parents, providing an important theoretical foundation for future identification of candidate wheat founder parents.

**Conclusions:**

These results reveal the genetic basis of founder parents and allele transmission for 87 agronomically important genes and shed light on breeding strategies for the next generation of elite founder parents in wheat.

**Supplementary Information:**

The online version contains supplementary material available at 10.1186/s12870-023-04098-x.

## Background

Wheat is one of the most economically important crops grown worldwide, with significant effects on the global economy and food security [1, 2]. Globally, China is the largest wheat producer and consumer. Wheat production and development are inseparable from the utilization of germplasm resources. Crop germplasm resources include landraces, modern cultivars, introduced varieties, and wild varieties. These germplasm resources carry a variety of genes and have immense value in crop breeding. During the 70-year wheat breeding history of China, breeders have perceived that some germplasm accessions possess excellent comprehensive traits, unique characteristics in prominent traits, and no obvious disadvantages. In addition, such varieties also have high general combining ability, and the favorable chromosomal loci and segments they carry can be continuously transmitted in their derivatives [3, 4]. The probability of breeders preferring and selecting accessions from these varieties has therefore increased. Such accessions, referred to as "founder parents" by breeders, are important breeding resources and exist in most crops. Since the 1950s, China has bred and promoted more than 3,000 wheat cultivars, more than 2,000 of which were bred from founder parents [4]. In parental selection, the utilization of founder parents can significantly improve breeding efficiency and provide an ideal genetic background for the improvement of new varieties [5].

Wheat founder parents have played important roles during different breeding time periods, and the utilization of founder parents reflects the direction of breeding selection. Using simple sequence repeats (SSRs) and other molecular markers to analyze founder parents and their derivatives, founder parents have been shown to have high genetic diversity, to carry genetic segments that are highly related to agronomically important traits, and to have conservative delivery of these traits in their derivatives [6–16]. The emergence of high-density single nucleotide polymorphism (SNP) arrays has enabled researchers to determine the detailed haplotype structure and genetic basis of trait variations [17]. SNP chips have also been widely used in the study of founder parents in crops such as corn [18, 19] and wheat [20]. For example, using the Maize SNP50 chip, a genome-wide scan of the maize founder parent “HuangZaoSi” and its derivative lines was performed, resulting in identification of 15 conserved genetic regions enriched in yield-related loci in the founder parent genome [21]. In recent years, whole-genome resequencing has been widely used in analyzing the genetic basis of crop founder parents and in quantitative trait locus (QTL) mining [22, 23]. Combining pedigree and SNP information, resequencing of the cotton genome revealed identity by descent (IBD) segments transmitted by Ekangmian 9 in its derivatives, and found 28 QTLs related to boll weight, boll number, lint percentage, and fiber quality [24]. In wheat, the recent study indicated that there are multiple ancestral haplotype blocks with stable inheritance in the central region of the wheat chromosome, revealing the expansion pathway of wheat and the trend of modern wheat breeding [25]. This demonstrates the utility of using various genotyping technologies combined with pedigree information of the founder parents to analyze key loci or important conservatively transmitted genomic regions; such analyses not only deepen understanding of the genetic basis of founder parents, but also promote the overarching objective of breeding selection in crops [26, 27].

Systematically analyzing the genetic basis of founder parents allows for prediction of candidate founder parents at the genomic level. Strong positive selection of favorable haplotypes by breeders has decreased the genetic diversity of the selected regions in founder parents and their derivatives [28–30]. A recent study found that the region near the centromere of chromosomes in wheat founder parents Aimengniu and Xiaoyan 6 formed a relatively large haplotype that was transmitted conservatively in derivatives, whereas the chromosome ends were prone to recombination and replacement [31]. Therefore, it is particularly important to analyze the transmission of founder parent chromosomal end genes in their derivatives. To date, more than 100 cloned genes have been made available from wheat [32]. Most of these are key genes located at the ends of chromosomes and control agronomically important traits, including yield [33, 34], stress resistance [35, 36], quality [37], and adaptability [38]. They have primarily been obtained through homologous cloning and map-based cloning strategies. Cloning these genes provides a basis for increasing yield, environmental adaptation, quality, and broad-spectrum resistance in wheat [39]. However, the distribution of these functional genes in wheat germplasm, especially in founder parents, has remained unclear. Fortunately, the development of kompetitive allele-specific PCR (KASP) functional markers has made it possible to detect genetic variation in these important functional genes and to understand their transmission in wheat founder parents.

In this study, a total of 366 wheat germplasm were analyzed, comprising founder parents, widely grown cultivars, and derivatives of four representative founder parents, Abbondanza, St2422/464, Zhoumai 16, and Jimai 22. These accessions were used to investigate yield and adaptability-related agronomic traits; genotypes of all accessions were identified using 87 KASP markers from cloned genes for agronomically important traits including yield, quality, adaptability, and stress resistance. Differences in phenotypic and genetic composition between founder parents and widely grown cultivars were systematically analyzed, and the favorable alleles carried by founder parents in the 87 agronomically important genes were identified. Furthermore, the transmission frequency of favorable alleles in the derivatives of founder parents was studied. Taken together, this study clarifies the genetic nature of founder parents and provides the theoretical basis for cultivating the next generation of candidate founder parents in wheat.

## Materials and methods

### Plant materials and DNA extraction

A total of 366 wheat accessions were used in this study, comprising founder parents, widely grown cultivars, and derivatives of four specific founder parents. Twenty-eight accessions belonged to more than one of these groups. Of the 40 founder parents, there were five Chinese landraces, 27 modern Chinese cultivars, and eight introduced modern cultivars. The widely grown cultivars comprised 47 modern Chinese cultivars. The founder parents and widely grown cultivars were primarily accessions released over five time periods, namely pre-1960s, 1970s, 1980s, 1990s, and 2000s. Here, some modern cultivars released after the 2000s that featured heavily in wheat breeding and produced many derivatives were also classified as founder parents, consistent with previous reports (Additional file 1: Table S1) [4, 40]. The dataset also included a total of 297 derivatives of the four founder parents Abbondanza, St2422/464, Zhoumai 16, and Jimai 22 (Additional files 1, 17: Table S1, Fig. S7). Specifically, there were 125 Abbondanza derivatives, 114 St2422/464 derivatives, 37 Zhoumai 16 derivatives, and 21 Jimai 22 derivatives. These founder parents were selected because Abbondanza and St2422/464 were the most widely used founder parents in Chinese wheat breeding and production in the 1960s and 1970s; Zhoumai 16 and Jimai 22 were bred in the 2000s and played key roles as founder parents in wheat breeding. In addition, 9 upstream and downstream pedigree accessions of Abbondanza and St2422/464, and one upstream pedigree accession of Bima 4 were also included. All materials were conserved in the Institute of Crop Sciences, Chinese Academy of Agricultural Sciences (CAAS), and were obtained from Chinese Crop Germplasm Resources Information System (http://www.cgris.net/zhongzhidinggou/index.php).

All accessions were grown in greenhouse environments for DNA extraction. Genomic DNA was extracted from fresh leaf tissue of each individual using plant DNA extraction kits (Qiagen, Hilden, Germany). DNA was diluted to a concentration of 40 ng/μL for KASP assays.

### Phenotypic assessment and statistical analyses

Phenotyping of 11 agronomically important traits was performed at two different locations in 2019 and 2020: the Chinese Academy of Agricultural Sciences (CAAS) Xinxiang Experiment Station in Henan province (113.5°E, 35.2°N) and the CAAS-Shunyi Experiment Station, Beijing (116.3°E, 40.0°N). Except for the derivatives of Zhoumai 16 and Jimai 22, all other accessions were planted in these two experimental fields, and field management followed local practices. Each accession was planted in a 2 m four-row plot with 25 cm between rows, and 40 seeds were planted in each row. From the middle of each plot, ten plants of each accession were used to measure heading date (HD, measured in days), flowering date (FD, days), plant height (PH, cm), effective tiller number (ETN, number), spike length (SL, cm), spikelet number per spike (SN, number), kernel number per spike (KN, number), thousand-kernel weight (TKW, g), kernel length (KL, mm), kernel width (KW, mm), and kernel thickness (KT, mm). The scoring standards for phenotypic traits were the same for both years and both experimental sites.

Basic statistics including mean value, minimum value, and maximum value for each trait were calculated for all accessions using Excel software. The best linear unbiased predictor (BLUP) method [41] was used to represent the mean value of each trait in this study.

### KASP genotyping of functional genes

In this study, robust KASP assays derived from 87 cloned genes related to agronomically important traits in wheat were used for genotyping (Additional file 2: Table S2). The physical positions (Mb) of all cloned genes were determined using BLAST against Chinese Spring v1.0. Mapchart was used to visually map the physical positions [42] (Additional file 11: Fig. S1). For the KASP assays, sequence information for 33 and 22 of the 87 genes were obtained from previous studies by Rasheed et al. [43] and Zhao et al. [44], respectively. Twenty-seven KASP assays were provided by Rasheed A (unpublished). The remaining five were also obtained from previous studies [45–49] (Additional file 2: Table S2). These 87 alleles of agronomically important genes were defined as favorable based on their desirable traits for human demands and production in wheat breeding in China [50], whereas the alleles for vernalization and photoperiod genes were not considered because there were different selection directions in different ecological regions in China (Additional file 3: Table S3).

KASP assays were performed in 384-well fluorescent quantitative plates. KASP Low Mixture was ordered from the Laboratory of the Government Chemist (LGC) Group. Allele-specific primers were designed to carry standard 6-carboxy-fluorescein (FAM) (5' GAAGGTGACCAAAGTTCATGCT 3') and hexachlorofluorescein (HEX) (5' GAAGGTCGGAGTCAACGATT 3') at the 3' end of the target SNP. A common primer was designed so that the total amplicon length was less than 120 bp [43]. The primer mixture consisted of 46 µl ddH_2_O, 30 µl common primer (at 100 µM), and 12 µl each tail primer (at 100 µM). Each reaction system had a total volume of 5.0 μL, comprising 2.2 μL of 40 ng/μL DNA, 2.5 μL of 1 × KASP V4.0 2 × Master mix (KBS-1016–017), 0.04 μL of MgCl_2_, 0.056 μL of primer mixture, and 0.204 μL of ddH_2_O. The PCR program was as follows: hot start at 95 ℃ for 15 min, followed by ten touchdown cycles (95 ℃ for 20 s; touchdown initially at 65 ℃ and decreasing by -1 ℃ per cycle of 25 s), followed by 30 additional cycles of annealing (95 ℃ for 10 s; 57 ℃ for 60 s). The amplified products were genotyped using the QuantStudio™ 7 Flex (Applied Biosystems by Life Technologies, San Diego, CA, USA), and the data were visualized using the associated QuantStudio™ Real-time PCR software (v.1.3) (Additional file 12: Fig. S2).

### Population structure and estimation of genetic differentiation

Nei's distances [51] were used to calculate genetic distances between the 366 accessions based on the 87 agronomically important genes. MEGA5 was used to construct and visualize the neighbor-joining tree [52]. SPSS Statistics v24.0 (Chicago, IL, USA) was used to calculate the Euclidean distance between accessions based on phenotypic data and to visualize the resulting phylogenetic tree of the founder parents and widely grown cultivars. TASSEL5 software was used to perform principal component analysis (PCA) on all accessions [53]. PCA plots were displayed and annotated with Origin 2018 software (https://www.originlab.com/). Based on polymorphic genotyping data, genetic differentiation coefficients (*F*st) were measured for population differentiation using genetic distance [54]. Gene flow (Nm) was also calculated between founder parents and widely grown cultivars from different breeding periods [55]. POPGENE software was used to calculate *F*st and Nm [56].

### Genetic composition analysis and allelic transmission

Th eggplot2 package in R (https://cran.r-project.org/web/packages/ggplot2/vignettes/ggplot2-in-packages.html) was used to calculate the allele frequencies of the 87 key genes in the 40 founder parents and 47 widely grown cultivars. The transmission plots of 87 alleles in the upstream and downstream pedigree cultivars of the founder parents Abbondanza and St2422/464 were also visualized using ggplot2. Genetic effect of favorable alleles for yield and adaptability genes were evaluated using the TASSEL5 software [53]. The transmission frequency of alleles (i.e., the frequency with which alleles carried by the founder parents Abbondanza, St2422/464, Zhoumai 16, or Jimai 22 were conserved in their derivatives) were represented by circos plots. Allele frequency was calculated with Excel 2013. The circos plots were annotated and formatted with TBtools [57].

## Results

### Population structure of wheat founder parents and widely grown cultivars

**Fig. 1:**
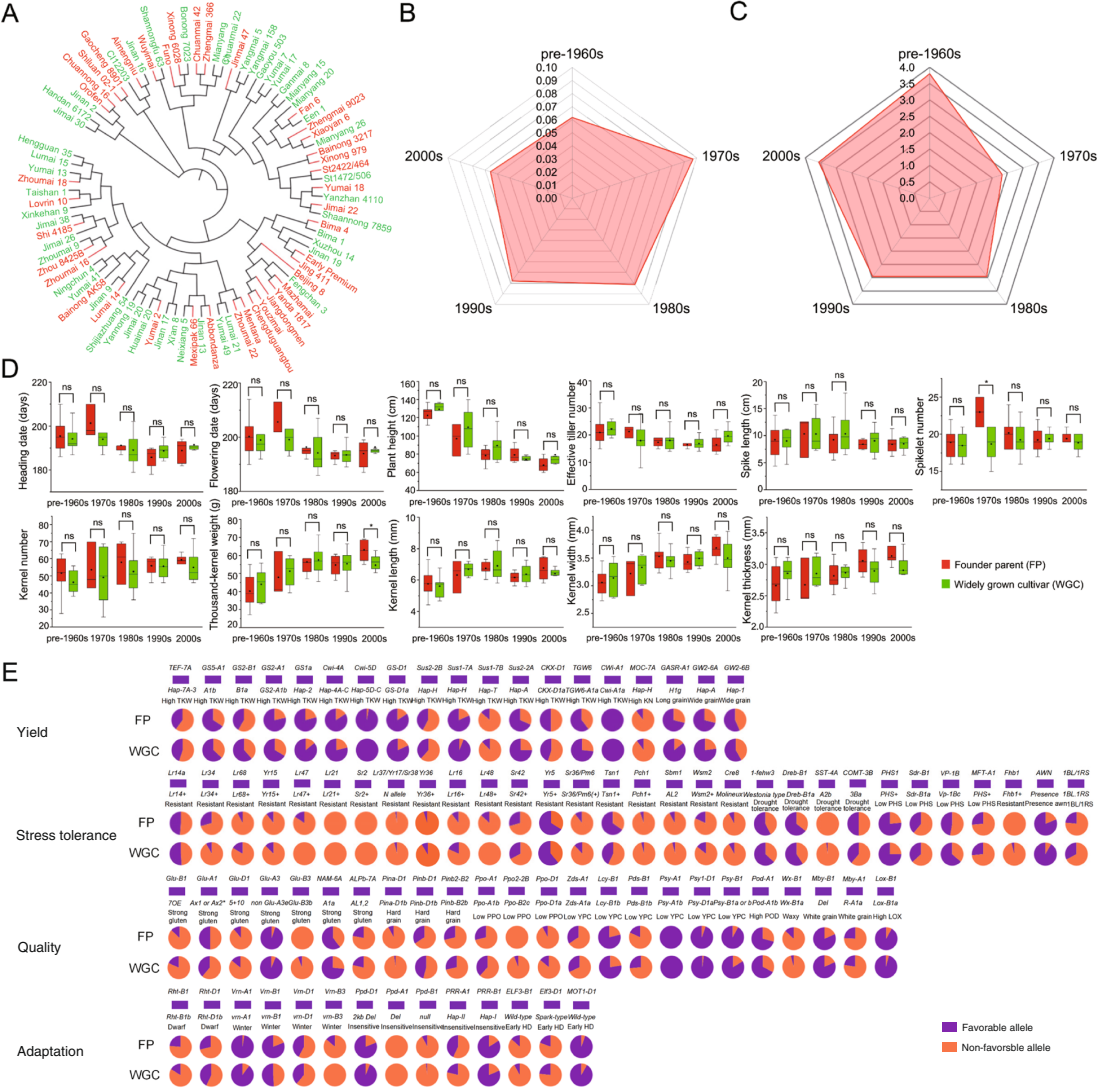
Phenotypic and genetic composition differences between founder parents and widely grown cultivars based on 11 agronomically important traits and 87 KASP markers. **A** A neighbor-joining tree of founder parents (FPs) and widely grown cultivars (WGCs) constructed using 87 KASP markers in agronomically important genes. Red and green represent FPs and WGCs, respectively. **B** Genetic differentiation coefficients (*F*st) between FPs and WGCs released from the pre-1960s period to the 2000s. **C** Gene flow between FPs and WGCs released from the pre-1960s period to the 2000s. **D** Phenotypic difference analysis of 11 agronomic traits between the FPs and WGCs in five breeding periods: pre-1960s, 1970s, 1980s, 1990s, and the 2000s. The traits analyzed were heading date (HD), flowering date (FD), plant height (PH), effective tiller number (ETN), spike length (SL), spikelet number (SN), kernel number (KN), thousand-kernel weight (TKW), kernel length (KL), kernel width (KW), and kernel thickness (KT). *P < 0.05; ns = not significant. **E** Allele frequencies of 87 agronomically important genes involved in grain yield, stress resistance, quality, and adaptability in both FPs and WGCs. The favorable and alternative alleles are shown in purple and orange, respectively

Phylogenetic tree construction and principal component analysis (PCA) were conducted using KASP markers for 87 agronomically important genes in 40 founder parents (FPs) and 47 widely grown cultivars (WGCs) (Additional files 1, 2, 3, 11, 12: Tables S1-S3, Figs. S1-S2). The FP and WGC pools clustered together with no obvious population structure, and the FPs were scattered throughout all branches of the WGCs, showing the crucial role of FPs in the WGC breeding process (Fig. 1A; Additional file 13: Fig. S3). These results were consistent with the previous study on fundamental roles of founder parents in wheat using SSR markers [5]. All accessions were categorized based on the time period in which they became widely used: pre-1960s, 1970s, 1980s, 1990s, or 2000s (Additional file 1: Table S1). The genetic differentiation coefficient (*F*st) between FPs and WGCs was the smallest in the pre-1960s period; the value peaked in the 1970s, then decreased gradually from the 1980s to the 2000s (Fig. 1B; Additional file 4: Table S4). Consistent with these results, gene flow between the FP and WGC pools was found to be highest in the pre-1960s period, to decrease to the smallest level in the 1970s, then to gradually increase from the 1980s to the 2000s (Fig. 1C; Additional file 4: Table S4).

Genetic composition analysis of the FPs at different time periods showed that all 40 FPs tended to carry favorable alleles in yield-related genes, but not in genes related to stress resistance or quality (Additional file 14: Fig. S4). For example, the favorable alleles *PHS* + for resistance-related genes have higher frequency among FPs at different stages. Among the quality genes, lower yellow pigment content alleles *Psy-A1b*, *Psy-B1a/b*, *Psy-D1a*, and *Lcy-B1b* were present in a high proportion of FPs at each stage. The strong gluten allele *non Glu-A3e*, white grain allele *Mby-B1-Del*, and higher enzyme activity allele *Lox-B1a* were also present in a very high proportion of FPs at different stages. After the 1970s, the FPs frequently carried the dwarf alleles *Rht-B1b* and *Rht-D1b*. These results indicated that, since the 1950s, the selection of FPs in the breeding process has been focused on high yield as the primary objective, but that other traits such as stripe rust resistance, pre-harvest sprouting, flour color, plant height, and adaptability have also undergone varying degrees of improvement in different time periods. The allele frequency of the 87 agronomically important genes in WGCs was similar to that of FPs (Fig. 1E; Additional file 15: Fig. S5).

Phenotypic analysis was also conducted to compare 11 yield- and adaptability-related traits between FPs and WGCs based on the best linear unbiased prediction (BLUP) values (Fig. 1D; Additional files 5, 6, 7, 16: Tables S5-S7, Fig. S6). This analysis further indicated that there were no significant phenotypic differences between FPs and WGCs in different time periods, and that trends in phenotypic changes over time were comparable between the two groups. More precisely, the heading date (HD), flowering date (FD), and plant height (PH) gradually decreased, but the thousand kernel weight (TKW), kernel length (KL), kernel width (KW), and kernel thickness (KT) increased over the decades. Thus, genetic composition analysis together with phenotypic trait comparison between the FPs and WGCs jointly demonstrate the primary reasons behind the lack of genetic structure among these elite wheat accessions. Altogether, these results are consistent with previous findings; they confirm that the genetic base of modern Chinese wheat cultivars has become very narrow, and that founder parents play a key role in current wheat breeding.

### Genetic composition of founder parents and widely grown cultivars from the same cross

**Fig. 2:**
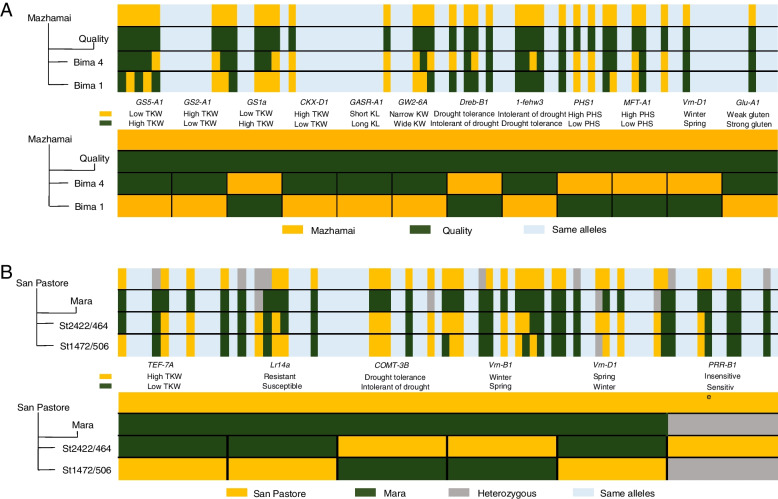
Allelic differences in 87 agronomically important genes between founder parents and widely grown cultivar siblings derived from the same hybrid combination. **A** Allelic differences in 87 agronomically important genes between Bima 4, the founder parent (FP), and Bima 1, the widely grown cultivar (WGC), which were derived from the same cross of Mazhamai/Quality. Of the 87 genes, 12 alleles referring to the lower panel were very different between Bima 4 and Bima 1, although 34 of the alleles differed between Mazhamai and Quality. Yellow and green vertical lines indicate alleles from Mazhamai and Quality, respectively. **B** Allelic differences in 87 agronomically important genes between St2422/464 (FP) and St1472/506 (WGC), which were derived from the same cross of San Pastore/Mara. Of the 87 genes, six alleles referring to the lower panel were very different between St2422/464 and St1472/506, although 40 alleles were very different between San Pastore and Mara. Yellow and green vertical lines indicate alleles from San Pastore and Mara, respectively; dark grey lines indicate heterozygosity

During the modern wheat breeding process in China, some crosses have produced founder parent and widely grown cultivar siblings, which have very different uses in wheat improvement. The genetic composition was compared between two sets of such sibling lines, Bima 4 and Bima 1 (from a Mazhamai/Quality cross) and St2422/464 and St1472/506 (from a San Pastore/Mara cross) (Fig. 2). The results showed that the founder parent Bima 4 and the widely grown cultivar Bima 1 shared 75 alleles out of the 87 agronomically important genes analyzed. Bima 4 carried favorable alleles for grain size (*GW2-6A-Hap-A* and *GASR-A1-H1c*), drought resistance (*Dreb-B1a* and *1-fehw3-Westonia*), and strong gluten (Glu-A1-Ax1orAx2*), whereas Bima 1 carried alleles for higher thousand-kernel weight (*GS2-A1b*, *GS1a-Hap-2*, and *CKX-D1a*) and pre-harvest sprouting (*PHS* + and *MFT-A1-PHS* +). Similarly, among the 87 agronomically important genes, the founder parent St2422/464 and the widely grown cultivar St1472/506 had 81 alleles in common. St2422/464 carried favorable alleles for leaf rust resistance (*Lr14* +), drought tolerance (*COMT-3Ba*), vernalization (*vrn-B1* and *vrn-D1*), and photoperiod insensitivity (*PRR-B1-Hap-I*), whereas St1472/506 carried alleles for higher thousand-kernel weight (*TEF-7A-Hap-3*). Taken together, these results suggest that favorable alleles in the widely grown cultivars are generally related to higher yield, whereas the founder parents have a comparatively richer genetic basis and, crucially, carry some specific favorable alleles in agronomically important genes.

### Genetic composition of representative founder parents in different breeding periods

Founder parents may carry their specific favorable alleles throughout different time periods due to the goals of the breeders. To explore the favorable alleles carried by founder parents and the transmission of such alleles to their derivatives, four representative founder parents released in different time periods were selected: Abbondanza (1960s), St2422/464 (1970s), Zhoumai 16 (2000s), and Jimai 22 (2000s) (Additional file 17: Fig. S7). Abbondanza has high yield, a thick stem, and resistance to stripe rust. It was planted as the main variety in the Yellow and Huai River valley winter wheat region in the 1960s. St2422/464 has short plant height, lodging resistance, rust resistance, and more flowers and kernels. In addition to immunity to the physiological races of stripe rust prevalent in the 1970s, St2422/464 has also been utilized in breeding as a dwarf source. Abbondanza and St2422/464 have been the most widely used wheat founder parents in the last century in China, and these two important parents have more derivatives than any of the other wheat founder parents studied here. Zhoumai 16 and Jimai 22 have played important roles in breeding and production as major founder parents in modern Chinese wheat breeding (i.e., the 2000s). Zhoumai 16 has short plant height, stalk rust resistance, and high general combining ability; Jimai 22 has high yield, good stability, wide adaptation capacity, and strong disease resistance. These four founder parents therefore represent the state of Chinese wheat breeding over different time periods.

**Fig. 3:**
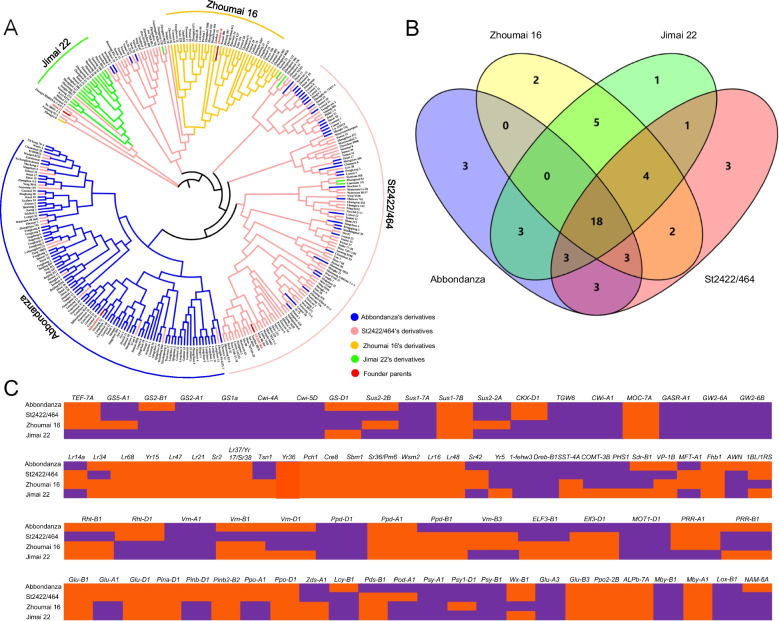
Genetic composition of four representative founder parents in 87 agronomically important genes. **A** A neighbor-joining tree of the four founder parents Abbondanza, St2422/464, Zhoumai 16, and Jimai 22, and derivatives of those accessions. The tree was constructed using 87 KASP markers in agronomically important genes. Derivatives of Abbondanza, St2422/464, Zhoumai 16, and Jimai 22 are shown in blue, pink, yellow, and green, respectively. The founder parents are shown in red. The four curves representing different subgroups of founder parents and their derivatives were drawn based on the genetic distance between them. **B** Common favorable alleles, uncommon favorable alleles, and specific favorable alleles of four founder parents in 87 agronomically important genes. **C** Genetic composition of the four representative founder parents in 87 agronomically important genes

Based on phylogenetic analysis (Fig. 3A) and PCA (Additional file 18: Fig. S8), the founder parents Abbondanza, St2422/464, Zhoumai 16, and Jimai 22 and their derivatives were clearly divided into four subgroups. Derivative accessions generally clustered together with their respective founder parents, suggesting that all four founder parents made large genetic contributions to their derivatives. There were obvious genetic differences among the derivatives of the founder parents in the previously mentioned 87 agronomically important genes.

Comparison of favorable alleles carried by the four founder parents revealed that, collectively, the majority of the favorable alleles were in yield-related genes: *GS2-A1b*, *GS1a-Hap-2*, *Cwi-4A-Hap-C*, *Cwi-5D-Hap-C*, *Sus1-7A-Hap-H*, *TGW6-A1a*, *Cwi-A1a*, *GASR-A1-H1c*, *GW2-6A-Hap-A*, and *GW2-6B-Hap-1* (Fig. 3B, C; Additional file 8: Table S8). This demonstrates that yield was historically an important objective in Chinese wheat breeding. They also carried two favorable alleles related to resistance (*1-fehw3-Westonia type*, and *Dreb-B1a*), four related to quality (*non Glu-A3e*, *Lox-B1a*, *Psy-A1b*, and *Psy-B1a/b*), and one related to early maturity (*MOT1-D1-Wild-type*). All four founder parents also carried specific favorable alleles for agronomically important traits. For example, founder parent Abbondanza carried favorable alleles related to lower pre-harvest sprouting (*Vp-1Bc*), lower yellow pigment content (*Pds-B1b*), and earlier flowering (*ELF3-D1-Spark-type*). St2422/464 carried favorable alleles related to leaf rust resistance (*Lr34* +), lower pre-harvest sprouting (*Sdr-B1a*), and reduced plant height (*Rht-B1b*). In the modern Chinese wheat founder parents, Jimai 22 carried the high thousand-kernel weight allele *TEF-7A-Hap-3*, and Zhoumai 16 carried the 1BL/1RS and the waxy allele *Wx-B1b*.

### Genetic contributions of founder parents to their derivatives in different breeding periods

**Fig. 4:**
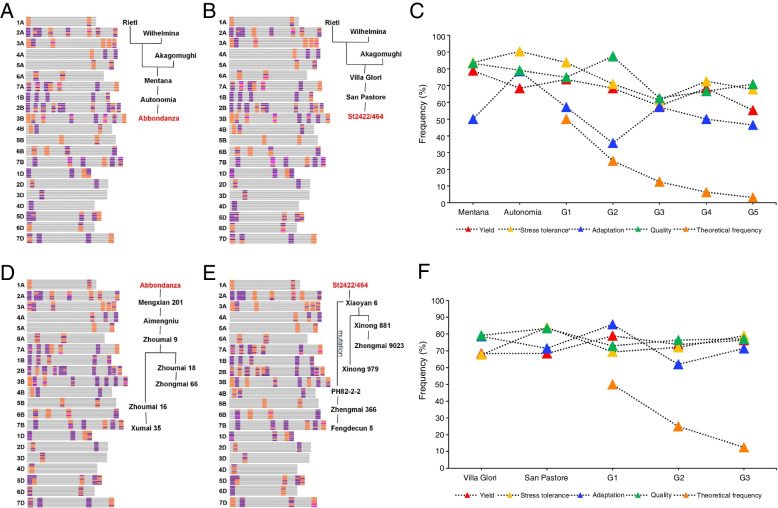
Allele transmission of founder parents Abbondanza and St2422/464 in parent and derivative accessions. **A**,** B** Allele transmission to founder parents Abbondanza and St2422/464 from their parents (Rieti/Wilhelmina//Akagomughi). The favorable and alternative alleles are shown in purple and orange, respectively, and the heterozygous types are shown in magenta. **C** Allele frequency in 87 agronomically important genes associated with grain yield, stress resistance, adaptability, and quality. Frequency is shown for the founder parent Abbondanza, its upstream parents, and several generations of its derivatives. **D**,** E** Allele transmission from founder parents Abbondanza and St2422/464 to their derivative accessions. The favorable and alternative alleles are shown in purple and orange, respectively, and the heterozygous types are shown in magenta. **F** Allele frequency in 87 agronomically important genes associated with grain yield, stress resistance, adaptability, and quality. Frequency is shown for the founder parent St2422/464, its upstream parents, and several generations of its derivatives

The genetic contributions of founder parents to their derivatives within different breeding periods were analyzed next. Tracing the upstream pedigree of founder parents Abbondanza (Fig. 4A) and St2422/464 (Fig. 4B), it was found that both were derived from the same famous cross combination, Rieti/Wilhemina//Akagomughi. Although the two founder parents inherited numerous alleles associated with yield, stress resistance, quality, and adaptation from their parents at a high frequency, they transmitted these alleles to their derivatives at an even higher frequency, which was also much higher than the theoretical frequency (Fig. 4C-F).

**Fig. 5:**
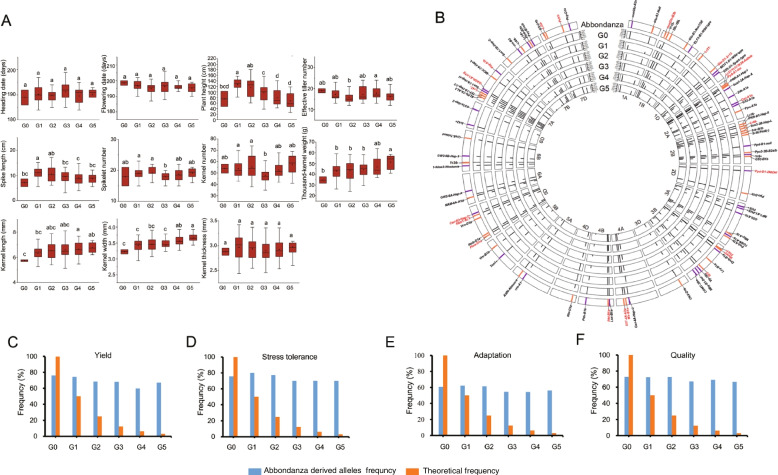
Phenotypic change tendency and frequency of alleles for 87 agronomically important genes in the derivatives of founder parent Abbondanza. **A** Phenotypic change tendency of 11 agronomic traits in different generations of the founder parent Abbondanza. Agronomic traits measured were heading date (HD), flowering date (FD), plant height (PH), effective tiller number (ETN), spike length (SL), spikelet number (SN), kernel number (KN), thousand-kernel weight (TKW), kernel length (KL), kernel width (KW), and kernel thickness (KT). The lowercase letters indicate statistically significant differences at *P* < 0.05. **B** Frequency of alleles derived from the founder parent Abbondanza in different generations of derivatives. The outermost circle shows alleles carried by Abbondanza for 87 agronomically important genes. From the outermost to the innermost circle, it shows different generations of Abbondanza: G0, G1, G2, G3, G4, and G5. The favorable and alternative alleles are shown in purple and orange, respectively. **C**-**F** Frequency of alleles for grain yield, stress resistance, adaptability, and quality derived from Abbondanza in different generations of derivatives. Light blue bars represent the frequency of Abbondanza-derived alleles and orange shows the theoretical frequency of alleles transmitted in the derivatives

Allelic transmission of the 87 agronomically important genes carried by the four founder parents was analyzed in depth using a large population of their derivatives. Phenotypic analysis of five generations of Abbondanza found that plant height gradually decreased in the derivatives, but thousand-kernel weight, grain length, and grain width increased significantly (Fig. 5A; Additional files 9, 19: Table S9, Fig. S9). This suggests that these traits were subjected to strong selection in different generations of the founder parent. Based on previous reports in the literature [8, 10], conservatively transmitted alleles were here defined as alleles carried by the founder parents with a transmission frequency of > 80% in each generation. Among the alleles for the 87 agronomically important genes, 22 alleles carried by Abbondanza were conservatively transmitted in the derivatives (Fig. 5B; Additional files 10, 20: Table S10, Fig. S10). Sibling lines of Abbondanza (G0) that carried the same alleles related to yield, quality, stress resistance, and adaptation transmitted these alleles at a lower rate than the theoretical frequency (Fig. 5C-F). This indicates that the sibling lines underwent recombination substitution at some loci and that their genetic composition differed significantly from that of Abbondanza. However, the transmission frequencies of Abbondanza alleles to the five derived generations (G1 to G5) were all above 60%, which was much higher than the theoretical frequency. This demonstrates that the founder parent Abbondanza had large genetic contributions to its derivatives, and that the derivatives inherited most alleles in the four trait categories from the founder parent. Analysis of the founder parent St2422/464 showed that its derivatives inherited its favorable alleles (Additional files 21, 22: Figs. S11A, S12) and continuously improved in phenotypic traits such as heading date, plant height, thousand-kernel weight, grain width, and grain thickness over the generations (Additional file 9: Table S9). Of the 87 agronomically important genes (Additional files 10, 21, 23: Table S10, Figs. S11B, S13), 27 alleles carried by St2422/464 were conservatively transmitted in the progeny, and the genetic contribution of St2422/464 to the derived generations far exceeded the theoretical frequency (Additional file 21: Fig. S11C-F).

Analysis of the founder parents Zhoumai 16 and Jimai 22, which were released in the 2000s, showed that up to 42 and 40 alleles, respectively, were conservatively transmitted in their derivatives. This indicates that more recent founder parents conservatively transmitted more alleles than accessions released in earlier eras did (Additional files 10, 24, 25: Table S10, Figs. S14, S15). Furthermore, Zhoumai 16 and Jimai 22 had higher genetic contributions to their derivatives in the four agronomic trait categories. Overall, this analysis showed that the four founder parents had their own specific characteristics and that throughout different breeding periods, all have had great genetic contributions to their derivatives at the individual gene level.

### Changing trends of favorable alleles conservatively transmitted by founder parents to their derivatives

The transmission patterns of alleles carried by Abbondanza, St2422/464, Zhoumai 16, and Jimai 22 to their derivatives were further comparatively analyzed (Additional files 26: Fig. S16). Specifically, the proportions of conservatively transmitted alleles (CTAs) and non-conservatively transmitted alleles (non-CTAs) in four founder parents from different breeding periods. The proportion of favorable and non-favorable alleles was also evaluated in CTAs and non-CTAs. The proportion of CTA in founder parents progressively increased from 25.3% to 48.3% over time, and the favorable alleles in CTA also increased from 13.6% to 45.0%. These results indicate that the genetic composition of founder parents was subject to continuous optimization during the wheat breeding process. However, based on the unfavorable alleles found in CTAs and non-CTAs in founder parents, these important germplasm resources still have a high percentage of defects in their genetic composition. Thus, there is still much room for their improvement through pyramiding of functional genes.

**Fig. 6:**
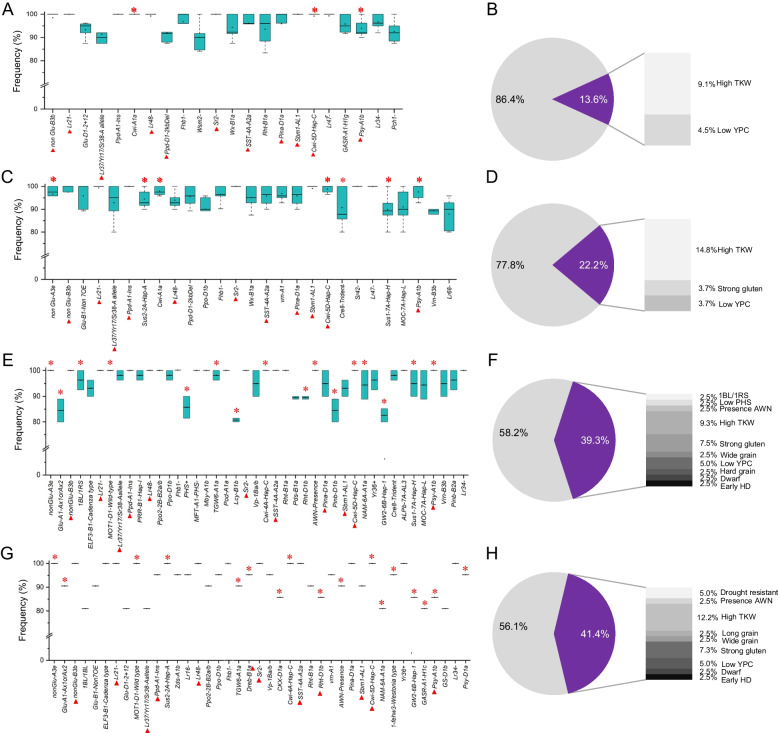
Alleles conservatively transmitted from four founder parents to their derivatives. **A**,** C**,** E**,** G** The transmission frequency of alleles conservatively transmitted from the founder parents Abbondanza, St2422/464, Zhoumai 16, and Jimai 22 to their derivatives. Favorable alleles are marked with red asterisks. The red triangles indicate alleles conservatively transmitted in derivatives of the founder parent Abbondanza. **B**,** D**,** F**,** H** The proportion of favorable and unfavorable alleles conservatively transmitted from the founder parents Abbondanza, St2422/464, Zhoumai 16, and Jimai 22 to their derivatives. The purple and grey sections represent the proportion of all favorable and unfavorable alleles, respectively, carried by founder parents. The proportions of favorable alleles associated with different agronomic traits from the four founder parents are shown from light to dark at the right

To clarify the types of CTAs in the four founder parents at different time periods, the proportion of CTAs and the phenotypic traits they influenced were further analyzed (Fig. 6). Eleven alleles were found to be common CTAs in the four founder parents, including two favorable alleles: higher thousand-kernel weight (*Cwi-5D-Hap-C*) and lower yellow pigment content (*Psy-A1b*). There were three favorable alleles conservatively transmitted in the derivatives of Abbondanza and six in the derivatives of St2422/464. Among these, the higher thousand-kernel weight alleles accounted for the highest proportion of favorable CTAs. There were 18 CTAs in Zhoumai 16; higher thousand-kernel weight accounted for the highest proportion of favorable CTAs, followed by alleles for stress resistance and good quality. Higher thousand-kernel weight alleles also accounted for the highest proportion of the 18 favorable CTAs in Jimai 22, followed by those for good quality and disease resistance. Collectively, these results indicate that the genetic composition of the representative founder parents was optimized as breeding progressed over time, with the number and types of favorable alleles carried gradually increasing and becoming enriched. Among the favorable alleles that have been strongly selected and conservatively transmitted, higher thousand-kernel weight has traditionally been the primary objective of breeding selection. However, additional favorable alleles related to good quality, stress resistance, and adaptability underwent strong selection and were retained. This implies that modern wheat breeding has put higher demands on quality, resistance, and adaptability based on continuous and sustainable yield improvement. However, the proportion of non-favorable CTAs in the four founder parents is still over 50%. This indicates the necessity of efforts to improve the genetic composition of modern founder parents; there is an urgent need to breed different elite founder parents with excellent genetic backgrounds and outstanding agronomic traits.

## Discussion

### Significant roles of founder parents in Chinese wheat breeding

Founder parents are widely considered to be excellent germplasm resources and have played important roles in the history of crop production and breeding. Elite Chinese landraces and introduced modern cultivars were initially used as founder parents, after which a large number of widely grown cultivars were commonly used in breeding, most of which were selected and bred using founder parents. Therefore, widely grown cultivars bred from founder parents had a high utilization rate, and were likely to become new founder parents themselves [58]. More significantly, Chinese landraces and modern Chinese cultivars in founder parents were also basically divided into two subgroups based on KASP markers (Fig. 1A), indicating the same results with a previous study [31].

However, not all widely grown cultivars can be used as founder parents in breeding. Generally, founder parents have high genetic diversity, high general combining ability, and unique excellent traits. Some founder parents and widely grown cultivars are sibling lines with very different utilization in breeding. For example, Li et al. [59] studied the relationship between Beijing 8 and its sibling lines based on phenotypes and SSR markers, and analysis of genetic differences found 59 specific alleles adjacent to the important trait genes in Beijing 8; some QTLs related to yield have also been found through genome comparison between Aimengniu and its sibling lines [60]. In the current study, genetic composition was compared at the individual gene level between Bima 1 and Bima 4 and between St1472/506 and St2422/464 (Fig. 2), which are sets of sibling lines each containing one founder parent and one widely grown cultivar. Founder parents were shown to have relatively high genetic diversity and to carry unique favorable alleles compared to their widely grown cultivar sibling lines. These results were consistent with previous findings [31, 61]. Key loci carried by founder parents are not only concentrated on a single trait, but offer advantages in multiple other traits, which is the biggest difference between founder parents and their widely grown cultivar siblings. Understanding the genetic basis of founder parents in this way can provide important information for future screening of novel candidate founder parents.

### Exploiting and utilizing the favorable alleles of founder parents at the gene level

With advances in molecular marker and next-generation sequencing technologies, genomics theories and methods have been applied to study the genetic basis of crop founder parents in depth. Recently, genotypic platforms, molecular markers, gene editing, genome selection, genome sequencing, and resequencing technologies have been used to comprehensively discover important genomic segments and key loci carried by founder parents in crops [62]. Notably, directly analyzing the genetic basis of the founder parents at the gene level can provide a deeper understanding of the favorable alleles carried by the founder parents, allowing researchers to intuitively find the advantages and disadvantages of founder parents. There have been more than 100 cloned genes in common wheat that have major effects on agronomically important traits [31, 63–65]. The traits controlled by these genes are primarily in four categories: yield, quality, stress resistance, and adaptability; however, some are related to domesticated genes or important traits such as fertility and resource utilization [66].

Kompetitive allele specific PCR (KASP) technology, due to its low cost, high throughput, high accuracy, and time-effectiveness, has been widely used for high-throughput detection of SNPs and insertion/deletion mutations in recent years, although it has the disadvantage of detecting only biallelic variation. Compared with traditional platforms, KASP is suitable for studying a small number of target sites in a large number of germplasm. In the present study, KASP functional markers derived from 87 agronomically important genes were used with a good genotyping effect in all the studied materials. Genetic analysis at the gene level showed that the founder parents carried more favorable alleles for disease resistance (*Lr34* + , *Lr47* + , and *Tsn1* +) and plant height (*Rht-B1b*) than the widely grown cultivars. Allele frequency analysis of the 87 functional genes in four representative founder parents (Abbondanza, St2422/464, Zhoumai 16, and Jimai 22) from different breeding periods indicated that the number of favorable alleles carried by these founder parents increased gradually over time (from 13.0% to 43.9%). Among favorable alleles that were strongly selected and conservatively transmitted, the allele for high thousand-kernel weight was consistently a target of selection over the course of decades. With the generation of improved modern varieties, many additional favorable alleles related to quality, stress resistance, and adaptability have been selected (Fig. 6). This shows that modern wheat breeding has focused on increasing yield while also improving quality, stress resistance, and adaptability.

### Allele enrichment maps in founder parents shed new light on breeding by design

Founder parents play extremely important roles in crop breeding, but the use of limited key parents leads to the problem of a narrow genetic basis for modern cultivars [67]. Therefore, analyzing the genetic basis of founder parents and their transmission rules in derivatives is of great significance for cultivating new breeding varieties and broadening the genetic basis of a new set of founder parents. In this study, based on 87 functional markers from agronomically important genes, analysis of the genetic basis of wheat founder parents showed that the main target of breeding during each time period has been to increase yield (Fig. 6). This is consistent with the results of previous studies [31, 68, 69]. Importantly, the analysis also showed that there are still a large number of favorable alleles in wheat founder parents that have not been fully utilized in breeding selection.

The 87 agronomically important genes were also used to generate favorable allele enrichment maps of the representative founder parents Abbondanza, St2422/464, Zhoumai 16, and Jimai 22 over different breeding periods (Additional file 27: Fig. S17). They were found to carry 34, 37, 39, and 36 favorable alleles, respectively, accounting for 39.1%, 42.5%, 44.8%, and 41.4% of the 87 genes. The results indicated that these founder parents all carried a relatively high number of favorable alleles and represented very good genetic composition overall. However, of the 87 genes analyzed, the non-favorable alleles carried by the four founder parents still accounted for a relatively high proportion (nearly 50%). Thus, to improve the breeding selection efficiency for yield, stress resistance, quality, and adaptability in wheat, it is necessary to further optimize the four types of agronomically important genes in the founder parents. The enrichment map demonstrated the advantages and disadvantages of each representative founder parent over different time periods at the gene level. Altogether, these results clarify important directions for further improvement of these founder parents and provide a theoretical basis for cultivating superior candidate founder parents in wheat. With the dramatic innovations taking place in genomics technologies, new founder parents with superior breeding values are expected to be available in the near future.

## Conclusions

The genetic composition characteristics of wheat founder parents were here analyzed using a population of 366 accessions comprising founder parents, widely grown cultivars, and the derivatives of four representative founder parents. Eighty-seven functional gene markers for yield, stress resistance, quality, and adaptability were used to comprehensively evaluate genetic composition and conservatively transmitted alleles. The results indicated that founder parents from different breeding periods possessed excellent performance in yield and had specific favorable alleles for agronomically important genes. These favorable alleles led to the cultivation of derivatives with strong transmission of excellent traits from the founder parents, although there were still many non-favorable alleles in the founder parents that were further improved over time. Allelic transmission analysis showed that yield has consistently been the primary selection objective of breeding, but that more favorable alleles for stress resistance, quality, and adaptability have been subject to selective pressure in modern wheat breeding. Altogether, these results reveal the genetic basis of founder parents and allele transmission for 87 agronomically important genes and shed light on breeding strategies for the next generation of elite founder parents in wheat.

## Supplementary Information

**Additional file 1: Table S1.** Detailed information and genotypic data for the 366 wheat accessions used in this study.

**Additional file 2: Table S2.** Basic information of alleles and primer sequences for the 87 KASP assays.

**Additional file 3: Table S3.** Favorable alleles for the 87 agronomically important genes.

**Additional file 4: Table S4.** Genetic differentiation coefficient (Fst) and gene flow (Nm) between founder parents (FPs) and widely grown cultivars (WGCs) during different breeding periods in 87 agronomically important genes.

**Additional file 5: Table S5.** Basic descriptive statistics for 11 agronomic traits in founder parents (FPs) and widely grown cultivars (WGCs).

**Additional file 6: Table S6.** Basic descriptive statistics for 11 agronomic traits in founder parents (FPs) and widely grown cultivars (WGCs) from different breeding periods.

**Additional file 7: Table S7.** Significance analysis for 11 agronomic traits between different breeding periods in founder parents (FPs) and widely grown cultivars (WGCs).

**Additional file 8: Table S8.** Common favorable alleles, uncommon favorable alleles, and specific favorable alleles carried by four founder parents in 87 agronomically important genes.

**Additional file 9: Table S9.** Basic descriptive statistics for 11 agronomic traits in the derivatives of founder parents Abbondanza and St2422/464.

**Additional file 10: Table S10.** Allele transmission frequency from founder parents Abbondanza, St2422/464, Zhoumai 16, and Jimai 22 to their derivatives in 87 agronomically important genes.

**Additional file 11: Figure S1. **Physical location of 87 agronomically important genes for grain yield, stress resistance, adaptability, and quality among 21 chromosomes in wheat. The left side of the chromosome indicates physical position (Mb) of each gene and the right shows gene names. Genes for grain yield, stress resistance, adaptability, and quality are represented by red triangles, yellow diamonds, dark blue squares, and green circles, respectively. The physical positions (Mb) of all genes were determined using BLAST against Chinese Spring v1.0.

**Additional file 12: Figure S2. **Scatter plots of eight genes identified using KASP genotyping. Red and blue dots show homozygous alleles, green and black dots represent heterozygous alleles and the negative control, respectively, and ‘x’ indicates missing types.

**Additional file 13: Figure S3. **Principal component analysis (PCA) of 40 founder parents and 47 widely grown cultivars based on 87 KASP markers in agronomically important genes. The founder parents (FPs) and widely grown cultivars (WGCs) are shown in red and green, respectively.

**Additional file 14: Figure S4. **Genetic composition and allele frequency of founder parents from different breeding periods. (A) Genetic composition of 87 yield, stress resistance, quality, and adaptability genes in 40 founder parents (FPs) from five different breeding periods. The favorable and alternative alleles are shown in purple and orange, respectively. Heterozygous and missing types are represented by magenta and black, respectively. (B) Allele frequencies of 87 agronomically important genes controlling yield, stress resistance, quality, and adaptability in 40 founder parents from five different breeding periods. The favorable and alternative alleles are shown in purple and orange, respectively.

**Additional file 15: Figure S5. **Genetic composition and allele frequency of widely grown cultivars from different breeding periods. (A) Genetic composition of 87 yield, resistance, quality, and adaptability genes in 47 widely grown cultivars (WGCs) from five different breeding periods. The favorable and alternative alleles are shown in purple and orange, respectively. Heterozygous types are shown in magenta and missing types are shown in black. (B) Allele frequencies of 87 agronomically important genes controlling grain yield, stress resistance, quality, and adaptability in 47 WGCs from five different breeding periods. The favorable and alternative alleles are shown in purple and orange, respectively.

**Additional file 16: Figure S6. **Phenotypic difference analysis between founder parents and widely grown cultivars from pre-1960s to 2000s in three environments. Founder parents (FPs) and widely grown cultivars (WGCs) are shown from five different breeding periods: pre-1960s, 1970s, 1980s, 1990s, and 2000s. Phenotypic traits investigated were heading date (A), flowering date (B), plant height (C), effective tiller number (D), spike length (E), spikelet number (F), kernel number (G), thousand-kernel weight (H), kernel length (I), kernel width (J), and kernel thickness (K). The three growth environments were Shunyi, Beijing in 2019 (2019SY), Xinxiang in Henan province in 2019 (2019XX), and Xinxiang in 2020 (2020XX). Red and green indicate FP and WGC, respectively.

**Additional file 17: Figure S7. **Family trees of four founder parents and derivative accessions based on pedigree information. (A) Family tree of the founder parent Abbondanza and its derivatives. Sibling lines (generation zero; G0), the first generation (G1), second generation (G2), third generation (G3), fourth generation (G4), and fifth generation (G5) are represented by light pink, light purple, light orange, light blue, light green, and dark green, respectively. (B) Family tree of the founder parent St2422/464 and its derivatives. The family tree includes generations from G0 to G5, which are indicated by color as described above. (C) Family tree of the founder parent Zhoumai 16 and its derivatives. The family tree includes generations G1 and G2, which are indicated by color as described above. (D) Family tree of the founder parent Jimai 22 and its derivatives. The family tree includes only generation G1, indicated by light purple.

**Additional file 18: Figure S8. **Principal component analysis (PCA) of the derivatives of four founder parents based on 87 KASP markers in agronomically important genes. The derivatives of founder parents Abbondanza, St2422/464, Zhoumai 16, and Jimai 22 are shown in dark blue, pink, yellow, and green, respectively; the founder parents are shown in red.

**Additional file 19: Figure S9. **Phenotypic difference analysis of 11 agronomic traits in the derivatives of founder parent Abbondanza in multiple environments. Different generations of Abbondaza include the sibling lines (generation zero, G0), first generation (G1), second generation (G2), third generation (G3), fourth generation (G4), and fifth generation (G5). Agronomic traits investigated were heading date (A), flowering date (B), plant height (C), effective tiller number (D), spike length (E), spikelet number (F), kernel number (G), thousand-kernel weight (H), kernel length (I), kernel width (J), and kernel thickness (K). The three growth environments were Shunyi, Beijing in 2019 (2019SY), Xinxiang in Henan province in 2019 (2019XX), and Xinxiang in 2020 (2020XX).

**Additional file 20: Figure S10. **Allele transmission from founder parent Abbondanza to its derivatives. The favorable and alternative alleles are shown in purple and orange, respectively. Heterozygous types are shown in magenta and missing types are shown in black. Conservatively transmitted alleles are labeled at the bottom of the figure, and red font indicates that the favorable allele was conservatively transmitted.

**Additional file 21: Figure S11. **Phenotypic change tendency and frequency of alleles in the derivatives of founder parent St2422/464. (A) Phenotypic change tendency of 11 agronomic traits in different generations of founder parent St2422/464. Agronomic traits investigated were heading date (HD), flowering date (FD), plant height (PH), effective tiller number (ETN), spike length (SL), spikelet number (SN), kernel number (KN), thousand-kernel weight (TKW), kernel length (KL), kernel width (KW), and kernel thickness (KT). Lowercase letters indicate statistically significant differences at p < 0.05. (B) Frequency of alleles derived from founder parent St2422/464 in its derivatives. The outermost circle shows alleles carried by St2422/464 for 87 agronomically important genes. From the outermost to the innermost circle, it shows different generations of St2422/464: G0, G1, G2, G3, G4, and G5. The favorable and alternative alleles are shown in purple and orange, respectively. (C)-(F) Frequency of alleles derived from St2422/464 for grain yield, stress tolerance, adaptation, and quality in the derivatives (G0-G5). Light blue bars represent the frequency of St2422/464-derived alleles and orange indicates the theoretical allele transmission frequency in derivatives of St2422/464.

**Additional file 22: Figure S12. **Phenotypic difference analysis of 11 agronomic traits in the derivatives of founder parent St2422/464 in multiple environments. Different generations of St2422/464 include the sibling lines (generation zero, G0), first generation (G1), second generation (G2), third generation (G3), fourth generation (G4), and fifth generation (G5). Agronomic traits investigated were heading date (A), flowering date (B), plant height (C), effective tiller number (D), spike length (E), spikelet number (F), kernel number (G), thousand-kernel weight (H), kernel length (I), kernel width (J), and kernel thickness (K). The three growth environments were Shunyi, Beijing in 2019 (2019SY), Xinxiang in Henan province in 2019 (2019XX), and Xinxiang in 2020 (2020XX).

**Additional file 23: Figure S13. **Allele transmission from founder parent St2422/464 to its derivatives. The favorable and alternative alleles are shown in purple and orange, respectively. Heterozygous types are shown in magenta and missing types are shown in black. Conservatively transmitted alleles are labeled at the bottom of the figure, and red font indicates that the favorable allele was conservatively transmitted.

**Additional file 24: Figure S14. **Allele transmission frequency in the derivatives of founder parent Zhoumai 16. (A) Distribution of alleles carried by founder parent Zhoumai 16 in its derivatives. The favorable and alternative alleles are shown in purple and orange, respectively, and red font indicates conservatively transmitted alleles. (B) Allele transmission from founder parent Zhoumai 16 to its derivatives. The favorable and alternative alleles are shown in purple and orange, respectively. Heterozygous types are shown in magenta and missing types are shown in black. Conservatively transmitted alleles are labeled at the bottom of the figure, and red font indicates that the favorable allele was conservatively transmitted. (C) Transmission frequency of alleles for grain yield, stress tolerance, adaptability, and quality derived from founder parent Zhoumai 16 to its derivatives. Light blue bars represent the frequency of Zhoumai 16-derived alleles and orange indicates the theoretical allele transmission frequency in the derivatives. G1: first generation; G2: second generation.

**Additional file 25: Figure S15. **Allele transmission frequency in the derivatives of founder parent Jimai 22. (A) Distribution of alleles carried by founder parent Jimai 22 in its derivatives. The favorable and alternative alleles are shown in purple and orange, respectively, and red font indicates conservatively transmitted alleles. (B) Allele transmission from founder parent Jimai 22 to its derivatives. The favorable and alternative alleles are shown in purple and orange, respectively. Heterozygous types are shown in magenta and missing types are shown in black. Conservatively transmitted alleles are labeled at the bottom of the figure, and red font indicates that the favorable allele was conservatively transmitted. (C) Transmission frequency of alleles for grain yield, stress tolerance, adaptability, and quality derived from founder parent Jimai 22 to its derivatives. Light blue bars represent the frequency of Jimai 22-derived alleles and orange indicates the theoretical allele transmission frequency in the derivatives. G1: first generation.

**Additional file 26: Figure S16. **Allele transmission frequency analysis from four founder parents to their derivatives in 87 agronomically important genes. (A) Frequency of allele transmission from founder parent Abbondanza to its derivatives in 87 agronomically important genes. The red dashed line indicates the threshold value for frequency of conservatively transmitted alleles. (B) The frequency of conservatively and non-conservatively transmitted alleles (non-CTAs) and the transmission frequency of favorable and non-favorable alleles from founder parent Abbondanza to its derivatives. Left: frequency of conservatively and non-conservatively transmitted alleles; middle: frequency of conservatively transmitted favorable and non-favorable alleles; right: frequency of non-conservatively transmitted favorable and non-favorable alleles. Conservatively transmitted alleles (CTAs) are shown in cyan, non-conservatively transmitted alleles (non-CTAs) are shown in gray, favorable alleles are shown in purple, and unfavorable alleles are shown in orange. (C) Frequency of allele transmission from founder parent St2422/464 to its derivatives in 87 agronomically important genes. The red dashed line indicates the threshold value for frequency of conservatively transmitted alleles. (D) The frequency of conservatively and non-conservatively transmitted alleles and the transmission frequency of favorable and non-favorable alleles from founder parent St2422/464 to its derivatives. Left: frequency of conservatively and non-conservatively transmitted alleles; middle: frequency of conservatively transmitted favorable and non-favorable alleles; right: frequency of non-conservatively transmitted favorable and non-favorable alleles. (E) Frequency of allele transmission from founder parent Zhoumai 16 to its derivatives in 87 agronomically important genes. The red dashed line indicates the threshold value for frequency of conservatively transmitted alleles. (F) The frequency of conservatively and non-conservatively transmitted alleles and the transmission frequency of favorable and non-favorable alleles from founder parent Zhoumai 16 to its derivatives. Left: frequency of conservatively and non-conservatively transmitted alleles; middle: frequency of conservatively transmitted favorable and non-favorable alleles; right: frequency of non-conservatively transmitted favorable and non-favorable alleles. (G) Frequency of allele transmission from founder parent Jiami 22 to its derivatives in 87 agronomically important genes. The red dashed line indicates the threshold value for frequency of conservatively transmitted alleles. (H) The frequency of conservatively and non-conservatively transmitted alleles and the transmission frequency of favorable and nonfavorable alleles from founder parent Jiami 22 to its derivatives. Left: frequency of conservatively and non-conservatively transmitted alleles; middle: frequency of conservatively transmitted favorable and non-favorable alleles; right: frequency of non-conservatively transmitted favorable and non-favorable alleles.

**Additional file 27: Figure S17. **Enrichment of favorable alleles for agronomically important genes in four founder parents. (A) Enrichment of favorable alleles for agronomically important genes in founder parent Abbondanza. Genes for yield, stress tolerance, adaptability, and quality are shown in red, yellow, blue, and green, respectively, distributed among chromosomes. The favorable and alternative alleles of each genes are shown in purple and orange, respectively. Favorable alleles of agronomically important genes are enriched on the middle chromosomes. (B) Enrichment of favorable alleles for agronomically important genes in founder parent St2422/464. (C) Enrichment of favorable alleles for agronomically important genes in founder parent Zhoumai 16. (D) Enrichment of favorable alleles for agronomically important genes in founder parent Jimai 22.

## Data Availability

All data generated or analysed during this study are included in this published article and its supplementary information files.
