# Supplementary material for: Conservatively transmitted alleles of key agronomic genes provide insights into the genetic basis of founder parents in bread wheat (Triticum aestivum L.)
Source: BMC Plant Biol. 2023 Feb 18;23:100. doi: 10.1186/s12870-023-04098-x (PMC9938602; doi:10.1186/s12870-023-04098-x)
Supplement: Supplementary file 11 — Additional file 11: Figure S1. Physical location of 87 agronomically important genes for grain yield, stress resistance, adaptability, and quality among 21 chromosomes in wheat. The left side of the chromosome indicates physical position (Mb) of each gene and the right shows gene names. Genes for grain yield, stress resistance, adaptability, and quality are represented by red triangles, yellow diamonds, dark blue squares, and green circles, respectively. The physical positions (Mb) of all genes were determined using BLAST against Chinese Spring v1.0. [file 12870_2023_4098_MOESM11_ESM.pdf]

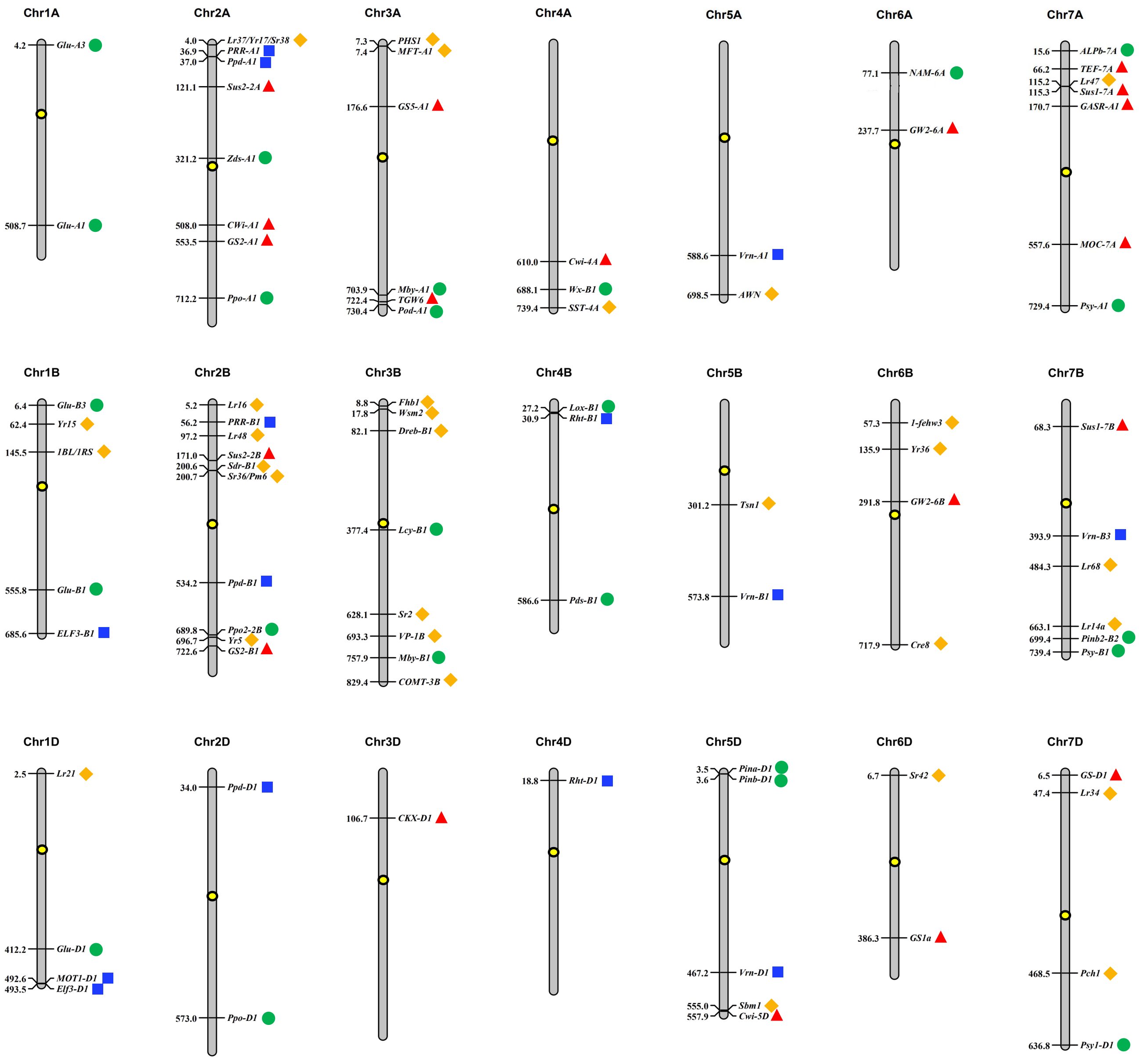


**Figure S1.** Physical location of 87 agronomically important genes for grain yield, stress resistance, adaptability, and quality among 21 chromosomes in wheat. The left side of the chromosome indicates physical position (Mb) of each gene and the right shows gene names. Genes for grain yield, stress resistance, adaptability, and quality are represented by red triangles, yellow diamonds, dark blue squares, and green circles, respectively. The physical positions (Mb) of all genes were determined using BLAST against Chinese Spring v1.0.
