# Supplementary material for: Conservatively transmitted alleles of key agronomic genes provide insights into the genetic basis of founder parents in bread wheat (Triticum aestivum L.)
Source: BMC Plant Biol. 2023 Feb 18;23:100. doi: 10.1186/s12870-023-04098-x (PMC9938602; doi:10.1186/s12870-023-04098-x)
Supplement: Supplementary file 12 — Additional file 12: Figure S2. Scatter plots of eight genes identified using KASP genotyping. Red and blue dots show homozygous alleles, green and black dots represent heterozygous alleles and the negative control, respectively, and ‘x’ indicates missing types. [file 12870_2023_4098_MOESM12_ESM.pdf]

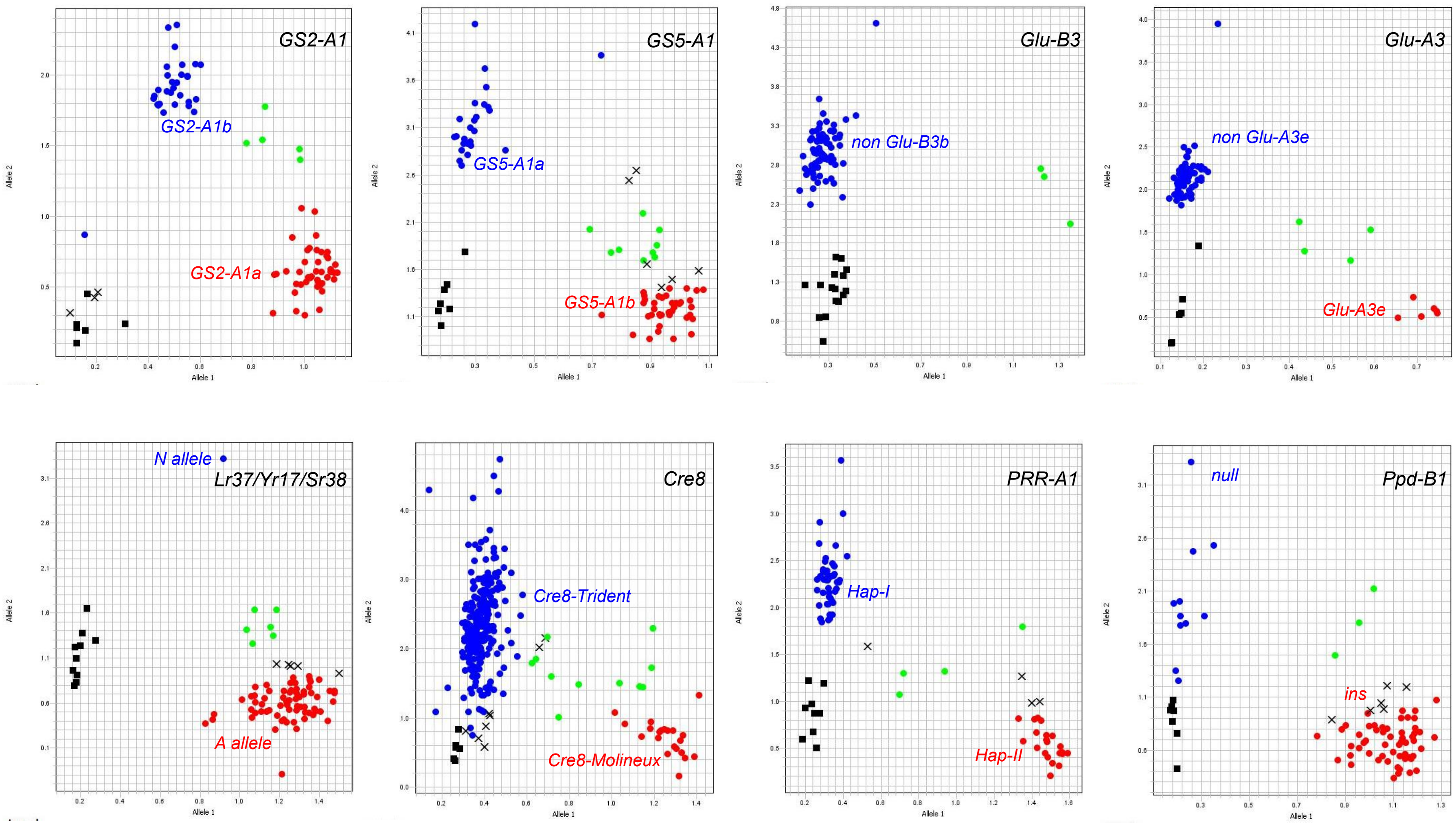

Figure S2. Scatter plots of eight genes identified using KASP genotyping. Red and blue dots show homozygous alleles, green and black dots represent heterozygous alleles and the negative control, respectively, and 'x' indicates missing types.
