# Supplementary material for: Conservatively transmitted alleles of key agronomic genes provide insights into the genetic basis of founder parents in bread wheat (Triticum aestivum L.)
Source: BMC Plant Biol. 2023 Feb 18;23:100. doi: 10.1186/s12870-023-04098-x (PMC9938602; doi:10.1186/s12870-023-04098-x)
Supplement: Supplementary file 13 — Additional file 13: Figure S3. Principal component analysis (PCA) of 40 founder parents and 47 widely grown cultivars based on 87 KASP markers in agronomically important genes. The founder parents (FPs) and widely grown cultivars (WGCs) are shown in red and green, respectively. [file 12870_2023_4098_MOESM13_ESM.pdf]

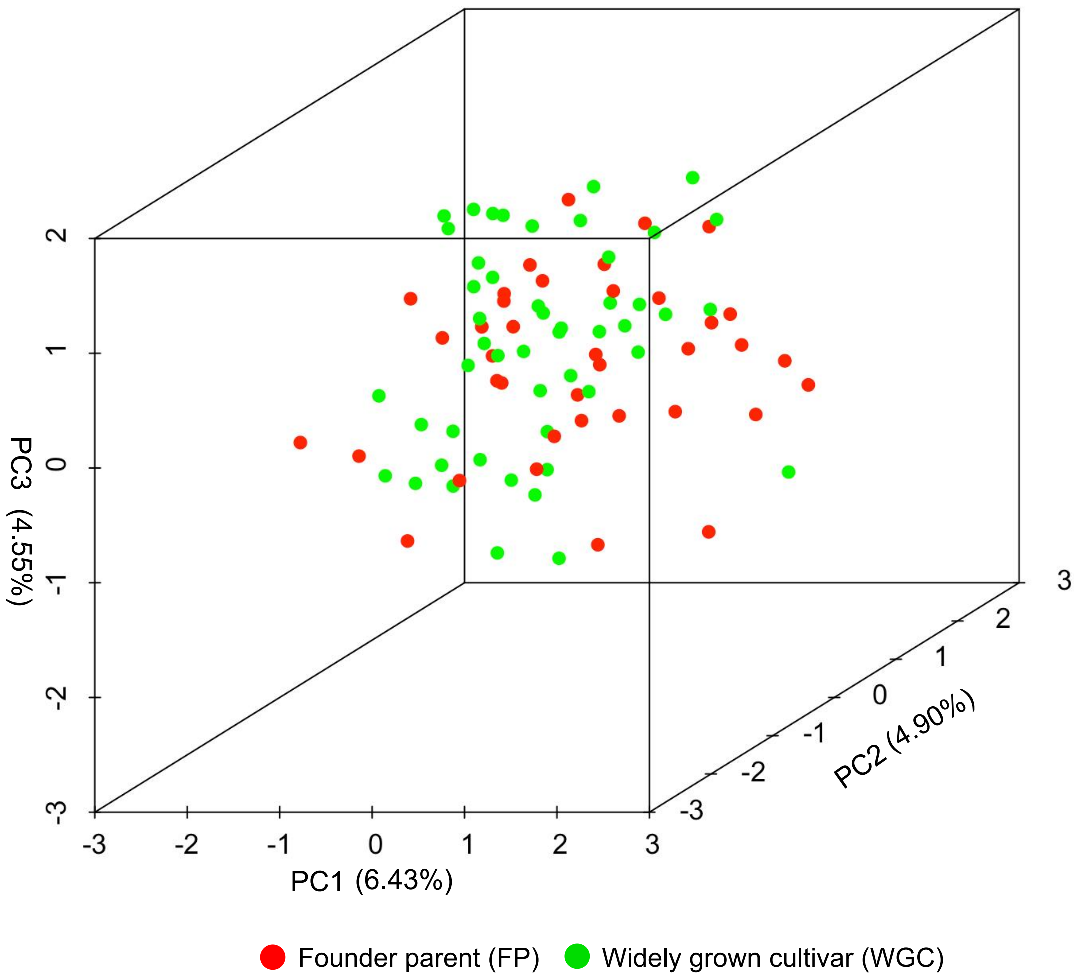

Figure S3. Principal component analysis (PCA) of 40 founder parents and 47 widely grown cultivars based on 87 KASP markers in agronomically important genes. The founder parents (FPs) and widely grown cultivars (WGCs) are shown in red and green, respectively.
