# Supplementary material for: Conservatively transmitted alleles of key agronomic genes provide insights into the genetic basis of founder parents in bread wheat (Triticum aestivum L.)
Source: BMC Plant Biol. 2023 Feb 18;23:100. doi: 10.1186/s12870-023-04098-x (PMC9938602; doi:10.1186/s12870-023-04098-x)
Supplement: Supplementary file 15 — Additional file 15: Figure S5. Genetic composition and allele frequency of widely grown cultivars from different breeding periods. (A) Genetic composition of 87 yield, resistance, quality, and adaptability genes in 47 widely grown cultivars (WGCs) from five different breeding periods. The favorable and alternative alleles are shown in purple and orange, respectively. Heterozygous types are shown in magenta and missing types are shown in black. (B) Allele frequencies of 87 agronomically important genes controlling grain yield, stress resistance, quality, and adaptability in 47 WGCs from five different breeding periods. The favorable and alternative alleles are shown in purple and orange, respectively. [file 12870_2023_4098_MOESM15_ESM.pdf]

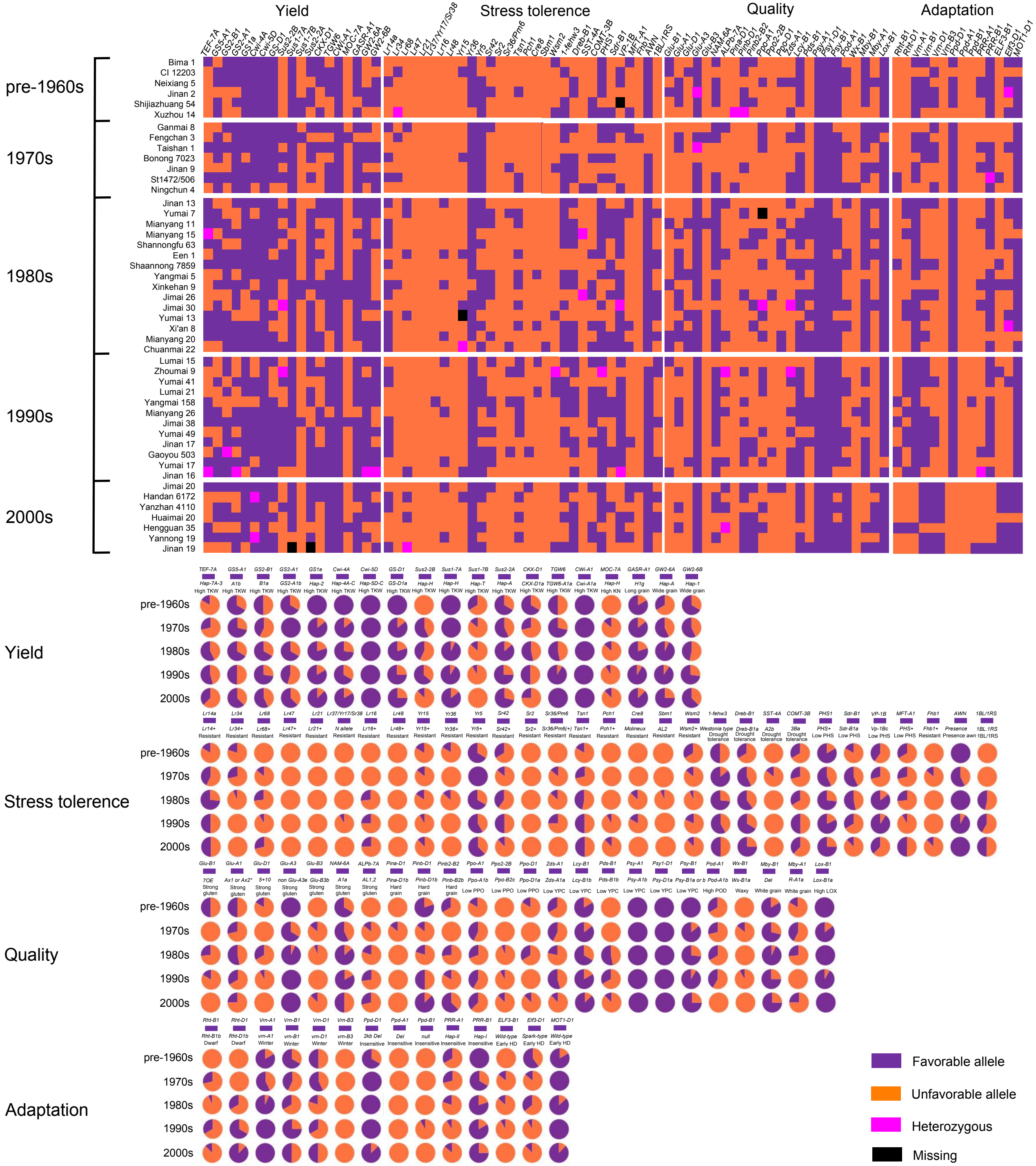

Figure S5. Genetic composition and allele frequency of widely grown cultivars from different breeding periods. (A) Genetic composition of 87 yield, resistance, quality, and adaptability genes in 47 widely grown cultivars (WGCs) from five different breeding periods. The favorable and alternative alleles are shown in purple and orange, respectively. Heterozygous types are shown in magenta and missing types are shown in black. (B) Allele frequencies of 87 agronomically important genes controlling grain yield, stress resistance, quality, and adaptability in 47 WGCs from five different breeding periods. The favorable and alternative alleles are shown in purple and orange, respectively.
