# Supplementary material for: Conservatively transmitted alleles of key agronomic genes provide insights into the genetic basis of founder parents in bread wheat (Triticum aestivum L.)
Source: BMC Plant Biol. 2023 Feb 18;23:100. doi: 10.1186/s12870-023-04098-x (PMC9938602; doi:10.1186/s12870-023-04098-x)
Supplement: Supplementary file 16 — Additional file 16: Figure S6. Phenotypic difference analysis between founder parents and widely grown cultivars from pre-1960s to 2000s in three environments. Founder parents (FPs) and widely grown cultivars (WGCs) are shown from five different breeding periods: pre-1960s, 1970s, 1980s, 1990s, and 2000s. Phenotypic traits investigated were heading date (A), flowering date (B), plant height (C), effective tiller number (D), spike length (E), spikelet number (F), kernel number (G), thousand-kernel weight (H), kernel length (I), kernel width (J), and kernel thickness (K). The three growth environments were Shunyi, Beijing in 2019 (2019SY), Xinxiang in Henan province in 2019 (2019XX), and Xinxiang in 2020 (2020XX). Red and green indicate FP and WGC, respectively. [file 12870_2023_4098_MOESM16_ESM.pdf]

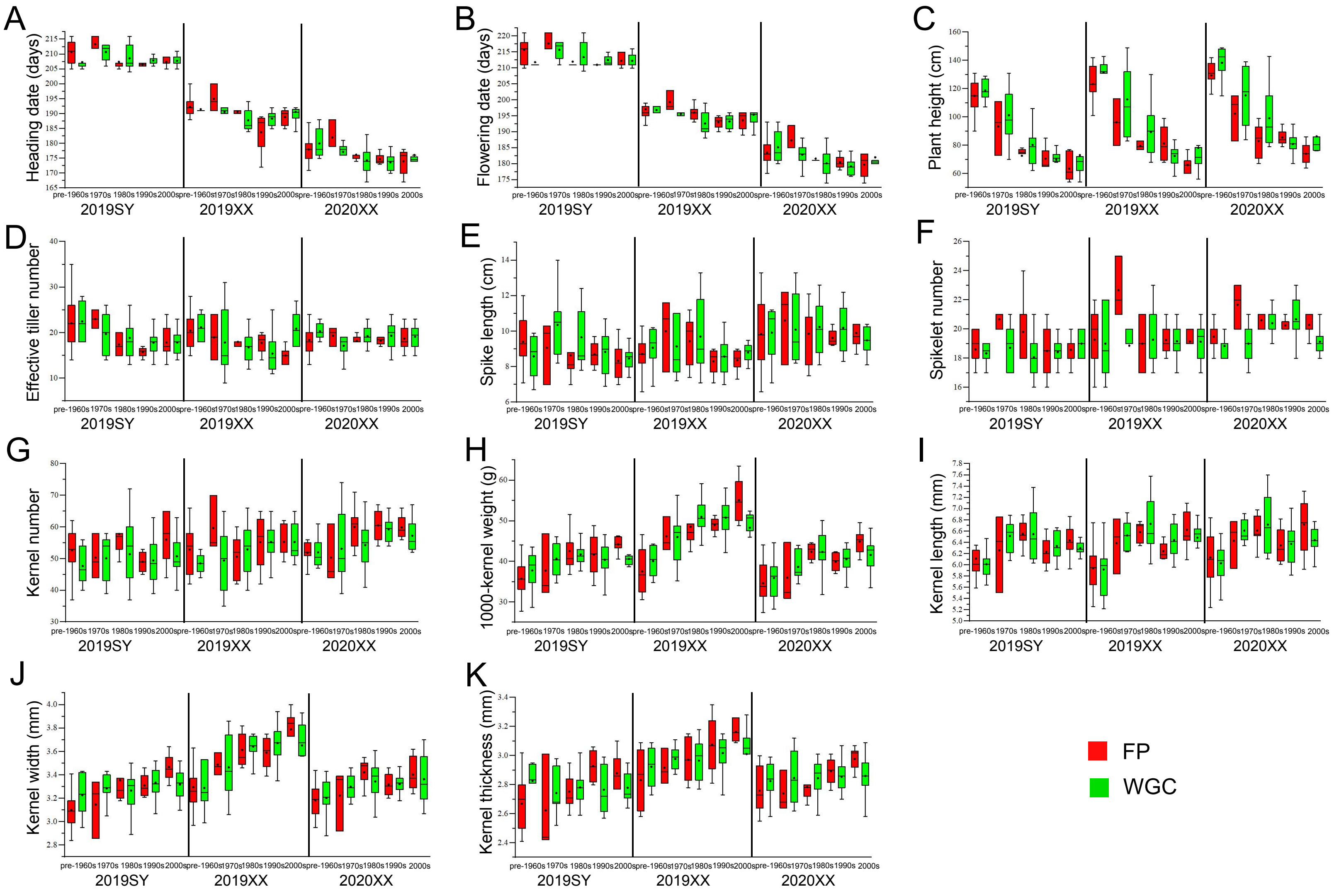

Figure S6. Phenotypic difference analysis between founder parents and widely grown cultivars from pre-1960s to 2000s in three environments. Founder parents (FPs) and widely grown cultivars (WGCs) are shown from five different breeding periods: pre-1960s, 1970s, 1980s, 1990s, and 2000s. Phenotypic traits investigated were heading date (A), flowering date (B), plant height (C), effective tiller number (D), spike length (E), spikelet number (F), kernel number (G), thousand-kernel weight (H), kernel length (I), kernel width (J), and kernel thickness (K). The three growth environments were Shunyi, Beijing in 2019 (2019SY), Xinxiang in Henan province in 2019 (2019XX), and Xinxiang in 2020 (2020XX). Red and green indicate FP and WGC, respectively.
