# Supplementary material for: Conservatively transmitted alleles of key agronomic genes provide insights into the genetic basis of founder parents in bread wheat (Triticum aestivum L.)
Source: BMC Plant Biol. 2023 Feb 18;23:100. doi: 10.1186/s12870-023-04098-x (PMC9938602; doi:10.1186/s12870-023-04098-x)
Supplement: Supplementary file 17 — Additional file 17: Figure S7. Family trees of four founder parents and derivative accessions based on pedigree information. (A) Family tree of the founder parent Abbondanza and its derivatives. Sibling lines (generation zero; G0), the first generation (G1), second generation (G2), third generation (G3), fourth generation (G4), and fifth generation (G5) are represented by light pink, light purple, light orange, light blue, light green, and dark green, respectively. (B) Family tree of the founder parent St2422/464 and its derivatives. The family tree includes generations from G0 to G5, which are indicated by color as described above. (C) Family tree of the founder parent Zhoumai 16 and its derivatives. The family tree includes generations G1 and G2, which are indicated by color as described above. (D) Family tree of the founder parent Jimai 22 and its derivatives. The family tree includes only generation G1, indicated by light purple. [file 12870_2023_4098_MOESM17_ESM.pdf]

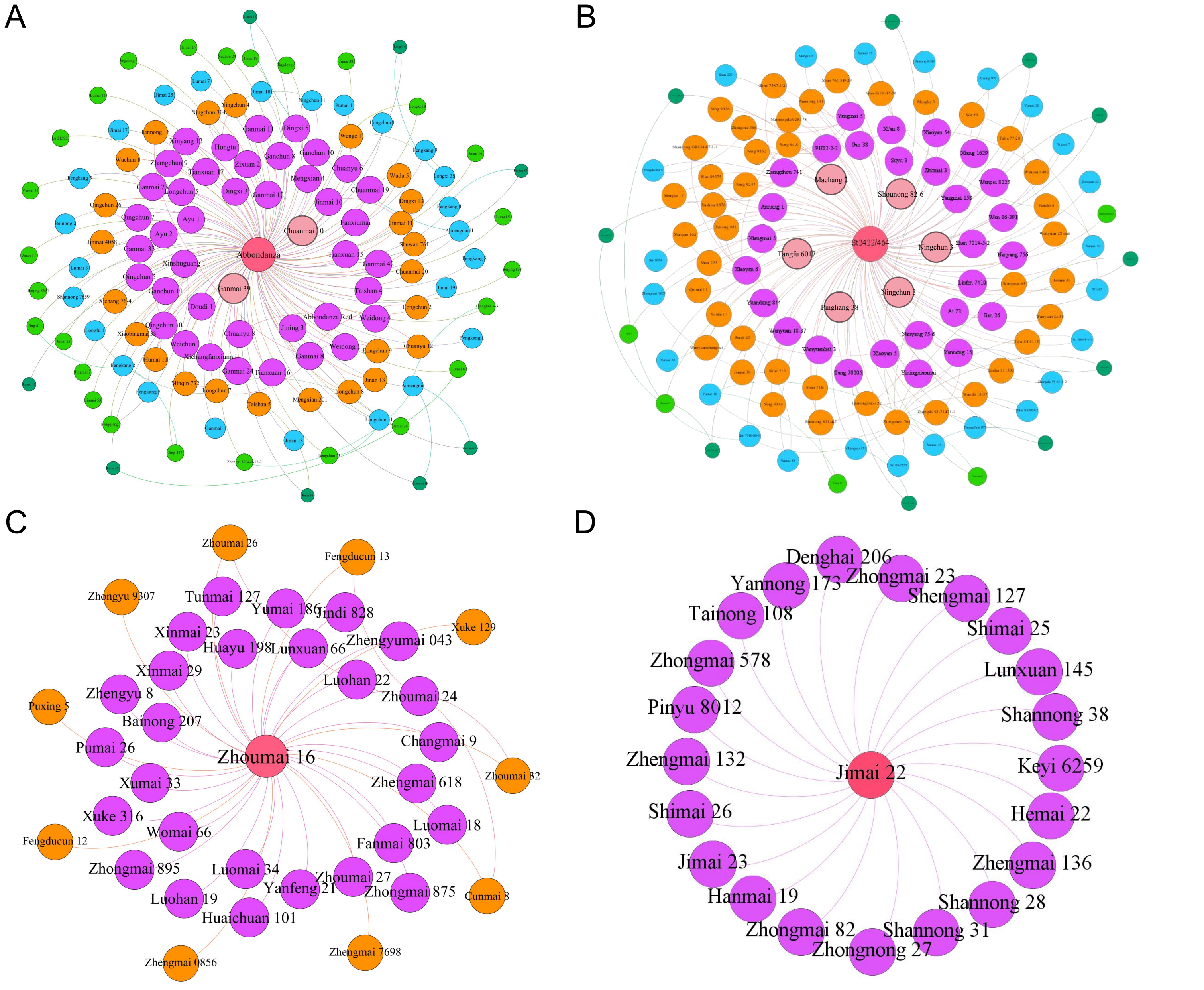

Figure S7. Family trees of four founder parents and derivative accessions based on pedigree information. (A) Family tree of the founder parent Abbondanza and its derivatives. Sibling lines (generation zero; G0), the first generation (G1), second generation (G2), third generation (G3), fourth generation (G4), and fifth generation (G5) are represented by light pink, light purple, light orange, light blue, light green, and dark green, respectively. (B) Family tree of the founder parent St2422/464 and its derivatives. The family tree includes generations from G0 to G5, which are indicated by color as described above. (C) Family tree of the founder parent Zhoumai 16 and its derivatives. The family tree includes generations G1 and G2, which are indicated by color as described above. (D) Family tree of the founder parent Jimai 22 and its derivatives. The family tree includes only generation G1, indicated by light purple.
