# Supplementary material for: Conservatively transmitted alleles of key agronomic genes provide insights into the genetic basis of founder parents in bread wheat (Triticum aestivum L.)
Source: BMC Plant Biol. 2023 Feb 18;23:100. doi: 10.1186/s12870-023-04098-x (PMC9938602; doi:10.1186/s12870-023-04098-x)
Supplement: Supplementary file 18 — Additional file 18: Figure S8. Principal component analysis (PCA) of the derivatives of four founder parents based on 87 KASP markers in agronomically important genes. The derivatives of founder parents Abbondanza, St2422/464, Zhoumai 16, and Jimai 22 are shown in dark blue, pink, yellow, and green, respectively; the founder parents are shown in red. [file 12870_2023_4098_MOESM18_ESM.pdf]

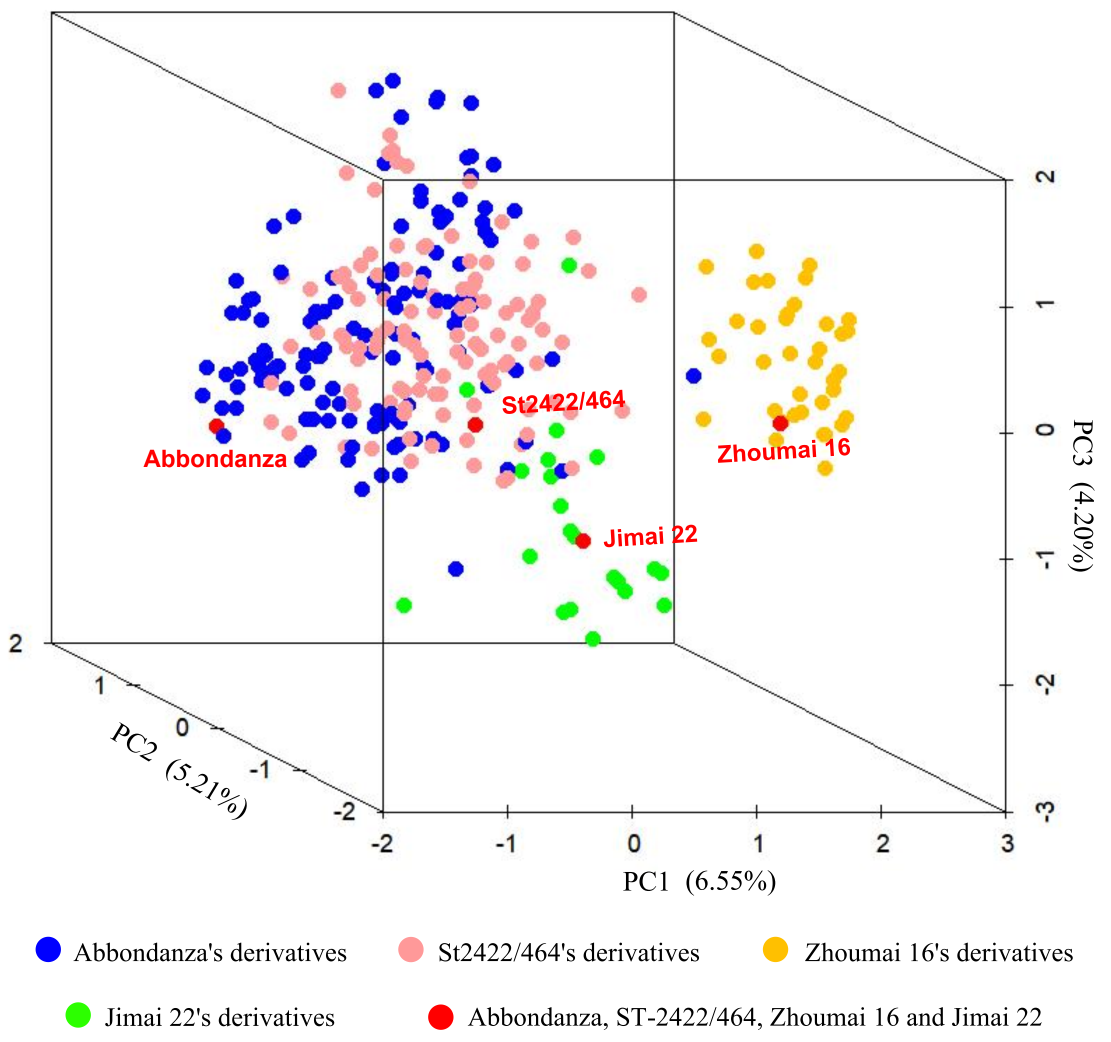

Figure S8. Principal component analysis (PCA) of the derivatives of four founder parents based on 87 KASP markers in agronomically important genes. The derivatives of founder parents Abbondanza, St2422/464, Zhoumai 16, and Jimai 22 are shown in dark blue, pink, yellow, and green, respectively; the founder parents are shown in red.
