# Supplementary material for: Conservatively transmitted alleles of key agronomic genes provide insights into the genetic basis of founder parents in bread wheat (Triticum aestivum L.)
Source: BMC Plant Biol. 2023 Feb 18;23:100. doi: 10.1186/s12870-023-04098-x (PMC9938602; doi:10.1186/s12870-023-04098-x)
Supplement: Supplementary file 20 — Additional file 20: Figure S10. Allele transmission from founder parent Abbondanza to its derivatives. The favorable and alternative alleles are shown in purple and orange, respectively. Heterozygous types are shown in magenta and missing types are shown in black. Conservatively transmitted alleles are labeled at the bottom of the figure, and red font indicates that the favorable allele was conservatively transmitted. [file 12870_2023_4098_MOESM20_ESM.pdf]

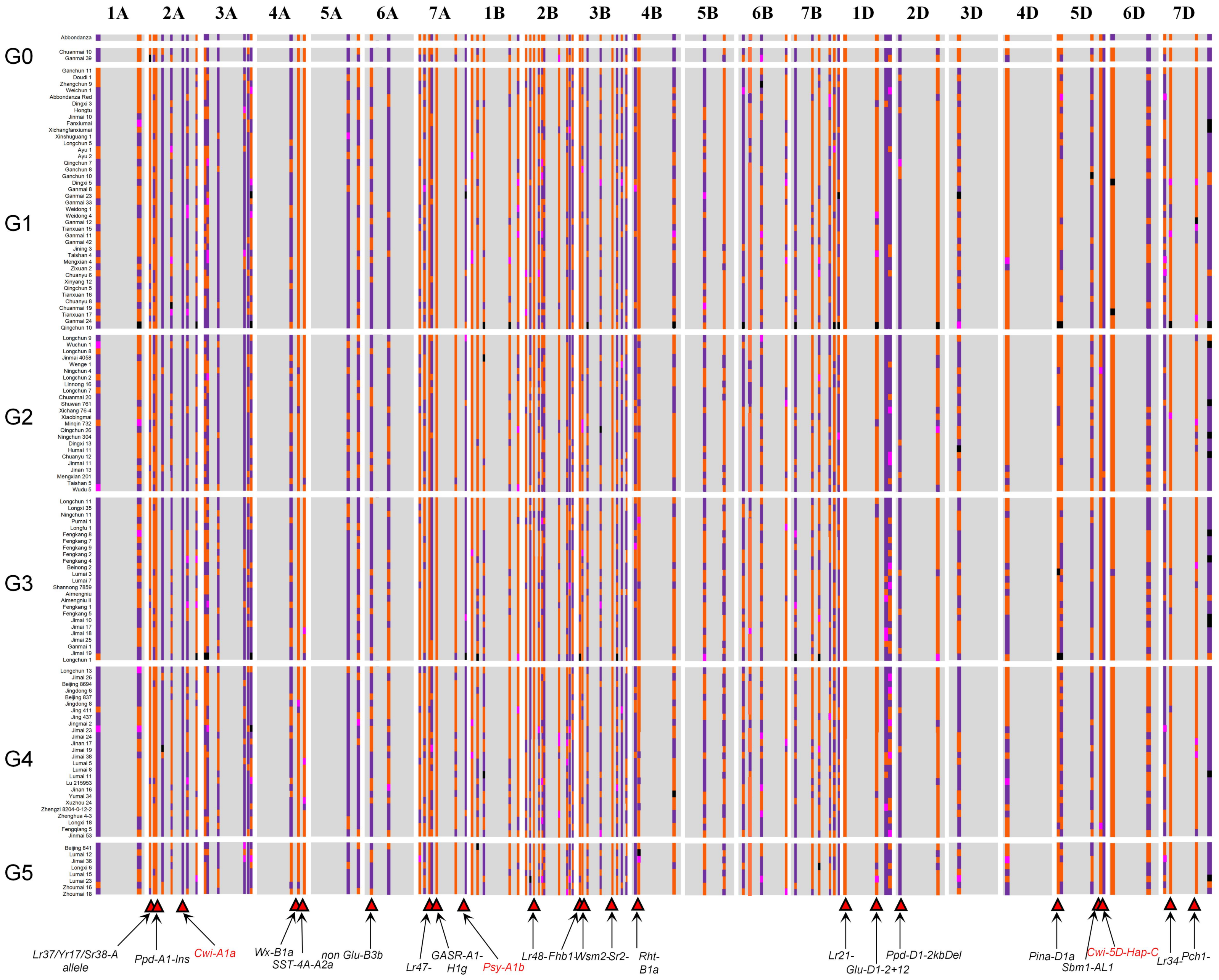

Figure S10. Allele transmission from founder parent Abbondanza to its derivatives. The favorable and alternative alleles are shown in purple and orange, respectively. Heterozygous types are shown in magenta and missing types are shown in black. Conservatively transmitted alleles are labeled at the bottom of the figure, and red font indicates that the favorable allele was conservatively transmitted.
