# Supplementary material for: Conservatively transmitted alleles of key agronomic genes provide insights into the genetic basis of founder parents in bread wheat (Triticum aestivum L.)
Source: BMC Plant Biol. 2023 Feb 18;23:100. doi: 10.1186/s12870-023-04098-x (PMC9938602; doi:10.1186/s12870-023-04098-x)
Supplement: Supplementary file 21 — Additional file 21: Figure S11. Phenotypic change tendency and frequency of alleles in the derivatives of founder parent St2422/464. (A) Phenotypic change tendency of 11 agronomic traits in different generations of founder parent St2422/464. Agronomic traits investigated were heading date (HD), flowering date (FD), plant height (PH), effective tiller number (ETN), spike length (SL), spikelet number (SN), kernel number (KN), thousand-kernel weight (TKW), kernel length (KL), kernel width (KW), and kernel thickness (KT). Lowercase letters indicate statistically significant differences at p < 0.05. (B) Frequency of alleles derived from founder parent St2422/464 in its derivatives. The outermost circle shows alleles carried by St2422/464 for 87 agronomically important genes. From the outermost to the innermost circle, it shows different generations of St2422/464: G0, G1, G2, G3, G4, and G5. The favorable and alternative alleles are shown in purple and orange, respectively. (C)-(F) Frequency of alleles derived from St2422/464 for grain yield, stress tolerance, adaptation, and quality in the derivatives (G0-G5). Light blue bars represent the frequency of St2422/464-derived alleles and orange indicates the theoretical allele transmission frequency in derivatives of St2422/464. [file 12870_2023_4098_MOESM21_ESM.pdf]

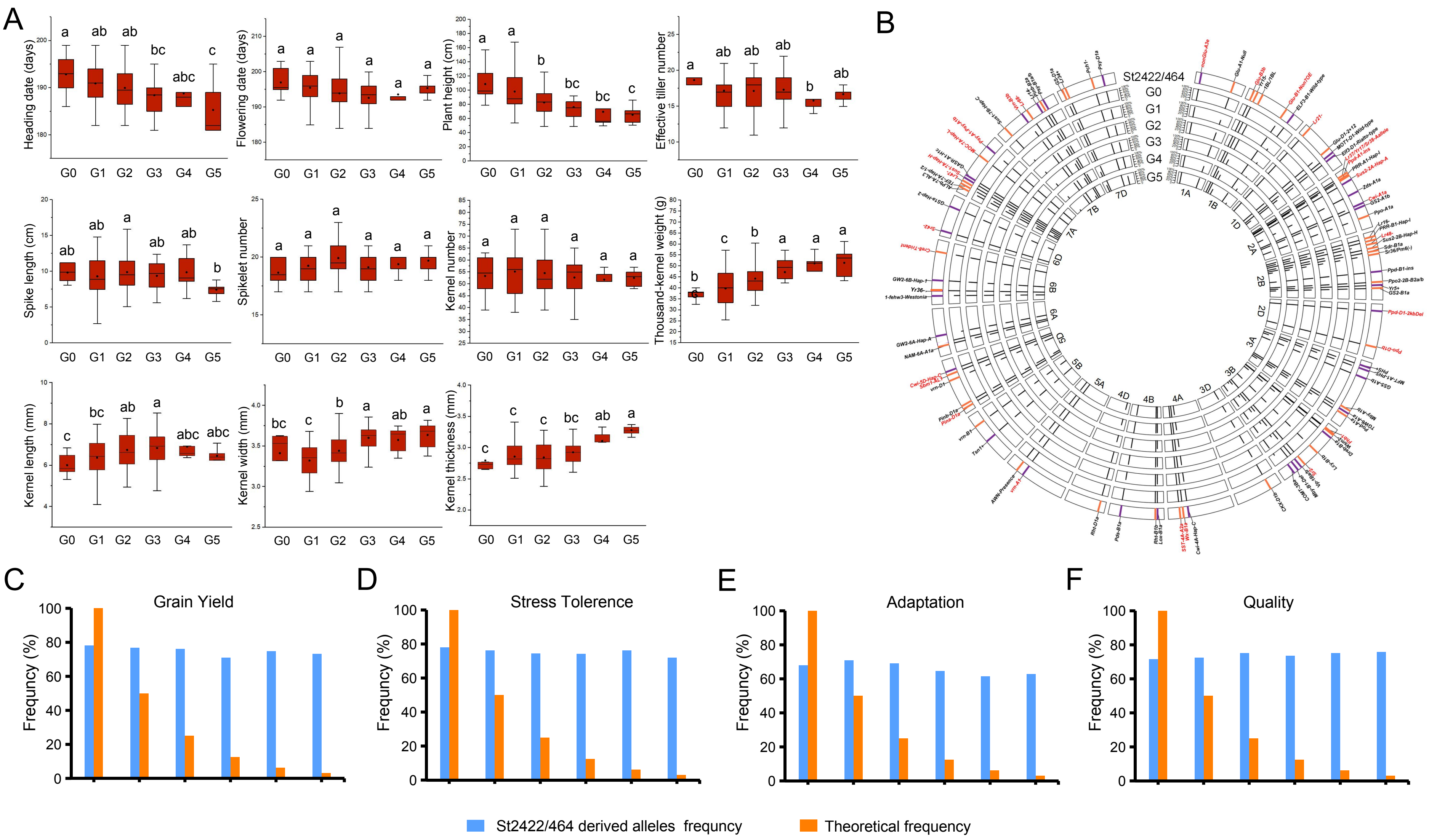

Figure S11. Phenotypic change tendency and frequency of alleles in the derivatives of founder parent St2422/464. (A) Phenotypic change tendency of 11 agronomic traits in different generations of founder parent St2422/464. Agronomic traits investigated were heading date (HD), flowering date (FD), plant height (PH), effective tiller number (ETN), spike length (SL), spikelet number (SN), kernel number (KN), thousand-kernel weight (TKW), kernel length (KL), kernel width (KW), and kernel thickness (KT). Lowercase letters indicate statistically significant differences at $p < 0.05$. (B) Frequency of alleles derived from founder parent St2422/464 in its derivatives. The outermost circle shows alleles carried by St2422/464 for 87 agronomically important genes. From the outermost to the innermost circle, it shows different generations of St2422/464: G0, G1, G2, G3, G4, and G5. The favorable and alternative alleles are shown in purple and orange, respectively. (C)-(F) Frequency of alleles derived from St2422/464 for grain yield, stress tolerance, adaptation, and quality in the derivatives (G0-G5). Light blue bars represent the frequency of St2422/464-derived alleles and orange indicates the theoretical allele transmission frequency in derivatives of St2422/464.
