# Supplementary material for: Conservatively transmitted alleles of key agronomic genes provide insights into the genetic basis of founder parents in bread wheat (Triticum aestivum L.)
Source: BMC Plant Biol. 2023 Feb 18;23:100. doi: 10.1186/s12870-023-04098-x (PMC9938602; doi:10.1186/s12870-023-04098-x)
Supplement: Supplementary file 22 — Additional file 22: Figure S12. Phenotypic difference analysis of 11 agronomic traits in the derivatives of founder parent St2422/464 in multiple environments. Different generations of St2422/464 include the sibling lines (generation zero, G0), first generation (G1), second generation (G2), third generation (G3), fourth generation (G4), and fifth generation (G5). Agronomic traits investigated were heading date (A), flowering date (B), plant height (C), effective tiller number (D), spike length (E), spikelet number (F), kernel number (G), thousand-kernel weight (H), kernel length (I), kernel width (J), and kernel thickness (K). The three growth environments were Shunyi, Beijing in 2019 (2019SY), Xinxiang in Henan province in 2019 (2019XX), and Xinxiang in 2020 (2020XX). [file 12870_2023_4098_MOESM22_ESM.pdf]

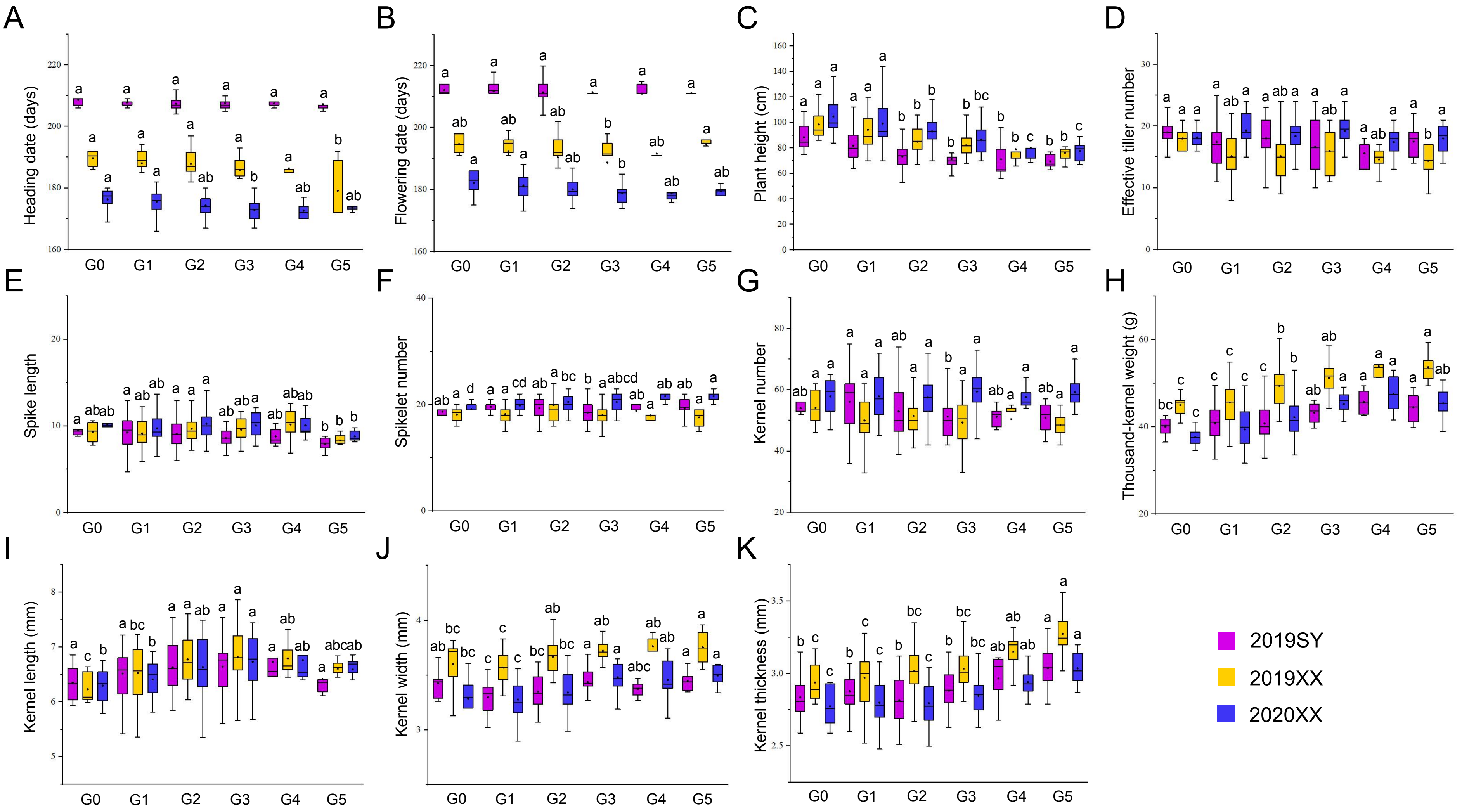

Figure S12. Phenotypic difference analysis of 11 agronomic traits in the derivatives of founder parent St2422/464 in multiple environments. Different generations of St2422/464 include the sibling lines (generation zero, G0), first generation (G1), second generation (G2), third generation (G3), fourth generation (G4), and fifth generation (G5). Agronomic traits investigated were heading date (A), flowering date (B), plant height (C), effective tiller number (D), spike length (E), spikelet number (F), kernel number (G), thousand-kernel weight (H), kernel length (I), kernel width (J), and kernel thickness (K). The three growth environments were Shunyi, Beijing in 2019 (2019SY), Xinxiang in Henan province in 2019 (2019XX), and Xinxiang in 2020 (2020XX).
