# Supplementary material for: Conservatively transmitted alleles of key agronomic genes provide insights into the genetic basis of founder parents in bread wheat (Triticum aestivum L.)
Source: BMC Plant Biol. 2023 Feb 18;23:100. doi: 10.1186/s12870-023-04098-x (PMC9938602; doi:10.1186/s12870-023-04098-x)
Supplement: Supplementary file 23 — Additional file 23: Figure S13. Allele transmission from founder parent St2422/464 to its derivatives. The favorable and alternative alleles are shown in purple and orange, respectively. Heterozygous types are shown in magenta and missing types are shown in black. Conservatively transmitted alleles are labeled at the bottom of the figure, and red font indicates that the favorable allele was conservatively transmitted. [file 12870_2023_4098_MOESM23_ESM.pdf]

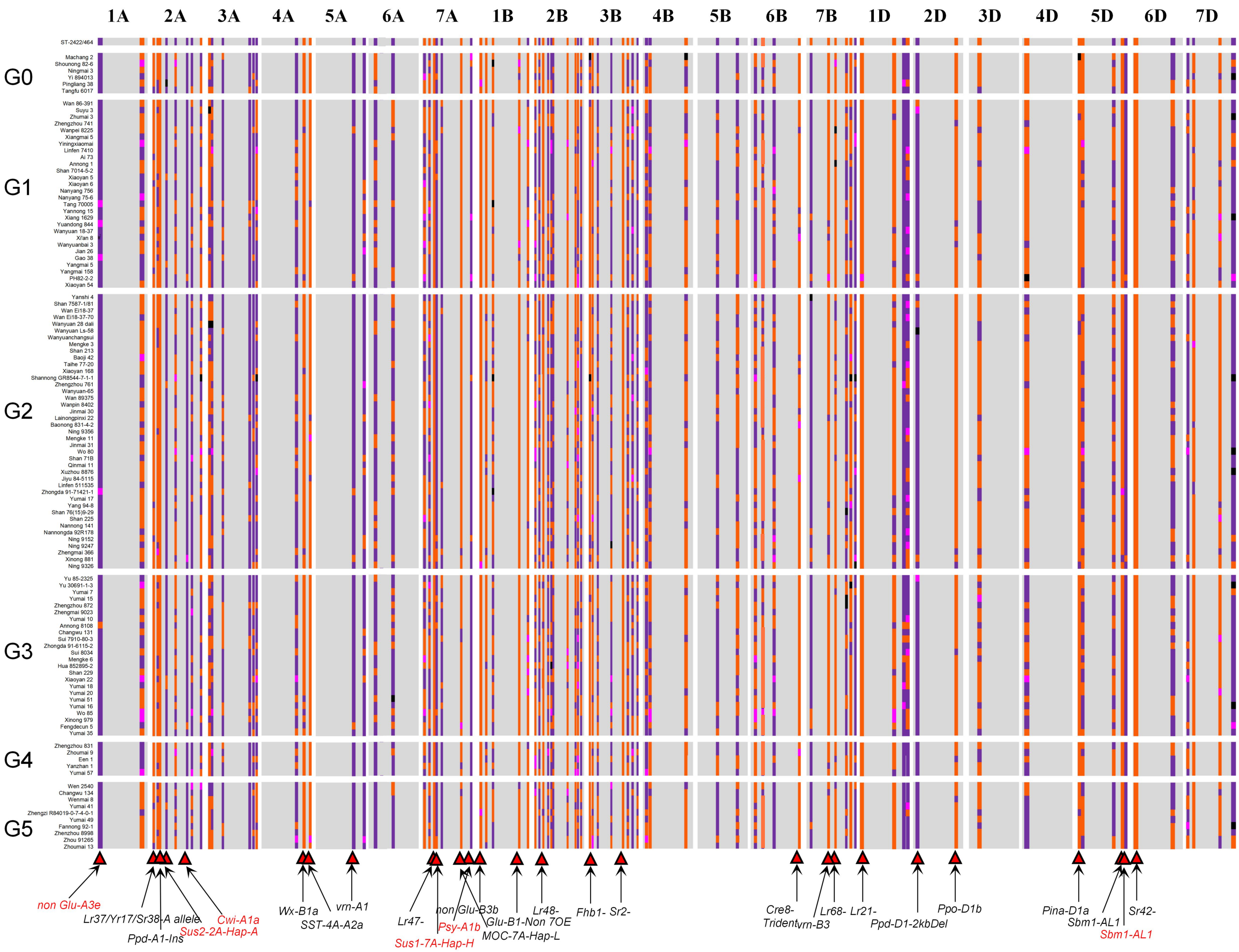

Figure S13. Allele transmission from founder parent St2422/464 to its derivatives. The favorable and alternative alleles are shown in purple and orange, respectively. Heterozygous types are shown in magenta and missing types are shown in black. Conservatively transmitted alleles are labeled at the bottom of the figure, and red font indicates that the favorable allele was conservatively transmitted.
