# Supplementary material for: Conservatively transmitted alleles of key agronomic genes provide insights into the genetic basis of founder parents in bread wheat (Triticum aestivum L.)
Source: BMC Plant Biol. 2023 Feb 18;23:100. doi: 10.1186/s12870-023-04098-x (PMC9938602; doi:10.1186/s12870-023-04098-x)
Supplement: Supplementary file 24 — Additional file 24: Figure S14. Allele transmission frequency in the derivatives of founder parent Zhoumai 16. (A) Distribution of alleles carried by founder parent Zhoumai 16 in its derivatives. The favorable and alternative alleles are shown in purple and orange, respectively, and red font indicates conservatively transmitted alleles. (B) Allele transmission from founder parent Zhoumai 16 to its derivatives. The favorable and alternative alleles are shown in purple and orange, respectively. Heterozygous types are shown in magenta and missing types are shown in black. Conservatively transmitted alleles are labeled at the bottom of the figure, and red font indicates that the favorable allele was conservatively transmitted. (C) Transmission frequency of alleles for grain yield, stress tolerance, adaptability, and quality derived from founder parent Zhoumai 16 to its derivatives. Light blue bars represent the frequency of Zhoumai 16-derived alleles and orange indicates the theoretical allele transmission frequency in the derivatives. G1: first generation; G2: second generation. [file 12870_2023_4098_MOESM24_ESM.pdf]

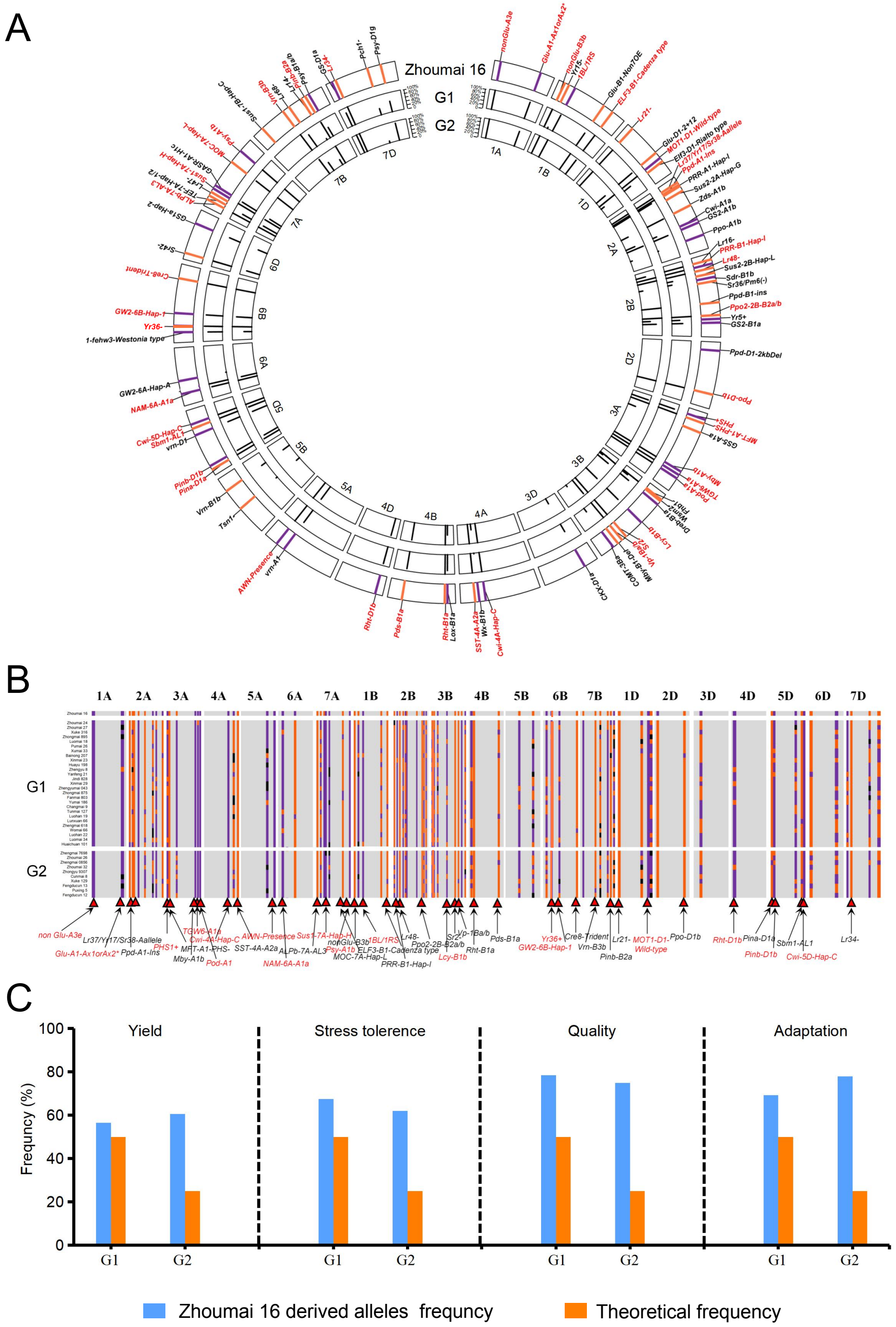


**Figure S14.** Allele transmission frequency in the derivatives of founder parent Zhoumai 16. (A) Distribution of alleles carried by founder parent Zhoumai 16 in its derivatives. The favorable and alternative alleles are shown in purple and orange, respectively, and red font indicates conservatively transmitted alleles. (B) Allele transmission from founder parent Zhoumai 16 to its derivatives. The favorable and alternative alleles are shown in purple and orange, respectively. Heterozygous types are shown in magenta and missing types are shown in black. Conservatively transmitted alleles are labeled at the bottom of the figure, and red font indicates that the favorable allele was conservatively transmitted. (C) Transmission frequency of alleles for grain yield, stress tolerance, adaptability, and quality derived from founder parent Zhoumai 16 to its derivatives. Light blue bars represent the frequency of Zhoumai 16-derived alleles and orange indicates the theoretical allele transmission frequency in the derivatives. G1: first generation; G2: second generation.
