# Supplementary figures and images for: Conservatively transmitted alleles of key agronomic genes provide insights into the genetic basis of founder parents in bread wheat (Triticum aestivum L.)
Source: BMC Plant Biol. 2023 Feb 18;23:100. doi: 10.1186/s12870-023-04098-x (PMC9938602; doi:10.1186/s12870-023-04098-x)

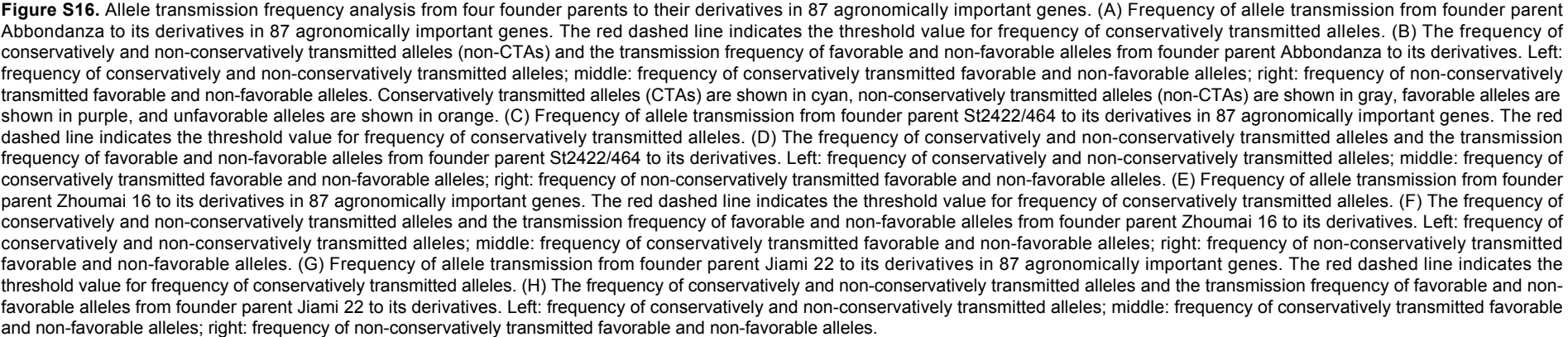

Supplement: Supplementary file 26 — Additional file 26: Figure S16. Allele transmission frequency analysis from four founder parents to their derivatives in 87 agronomically important genes. (A) Frequency of allele transmission from founder parent Abbondanza to its derivatives in 87 agronomically important genes. The red dashed line indicates the threshold value for frequency of conservatively transmitted alleles. (B) The frequency of conservatively and non-conservatively transmitted alleles (non-CTAs) and the transmission frequency of favorable and non-favorable alleles from founder parent Abbondanza to its derivatives. Left: frequency of conservatively and non-conservatively transmitted alleles; middle: frequency of conservatively transmitted favorable and non-favorable alleles; right: frequency of non-conservatively transmitted favorable and non-favorable alleles. Conservatively transmitted alleles (CTAs) are shown in cyan, non-conservatively transmitted alleles (non-CTAs) are shown in gray, favorable alleles are shown in purple, and unfavorable alleles are shown in orange. (C) Frequency of allele transmission from founder parent St2422/464 to its derivatives in 87 agronomically important genes. The red dashed line indicates the threshold value for frequency of conservatively transmitted alleles. (D) The frequency of conservatively and non-conservatively transmitted alleles and the transmission frequency of favorable and non-favorable alleles from founder parent St2422/464 to its derivatives. Left: frequency of conservatively and non-conservatively transmitted alleles; middle: frequency of conservatively transmitted favorable and non-favorable alleles; right: frequency of non-conservatively transmitted favorable and non-favorable alleles. (E) Frequency of allele transmission from founder parent Zhoumai 16 to its derivatives in 87 agronomically important genes. The red dashed line indicates the threshold value for frequency of conservatively transmitted alleles. (F) The frequency of conservatively a [file 12870_2023_4098_MOESM26_ESM.pdf]
