# Supplementary material for: Conservatively transmitted alleles of key agronomic genes provide insights into the genetic basis of founder parents in bread wheat (Triticum aestivum L.)
Source: BMC Plant Biol. 2023 Feb 18;23:100. doi: 10.1186/s12870-023-04098-x (PMC9938602; doi:10.1186/s12870-023-04098-x)
Supplement: Supplementary file 27 — Additional file 27: Figure S17. Enrichment of favorable alleles for agronomically important genes in four founder parents. (A) Enrichment of favorable alleles for agronomically important genes in founder parent Abbondanza. Genes for yield, stress tolerance, adaptability, and quality are shown in red, yellow, blue, and green, respectively, distributed among chromosomes. The favorable and alternative alleles of each genes are shown in purple and orange, respectively. Favorable alleles of agronomically important genes are enriched on the middle chromosomes. (B) Enrichment of favorable alleles for agronomically important genes in founder parent St2422/464. (C) Enrichment of favorable alleles for agronomically important genes in founder parent Zhoumai 16. (D) Enrichment of favorable alleles for agronomically important genes in founder parent Jimai 22. [file 12870_2023_4098_MOESM27_ESM.pdf]

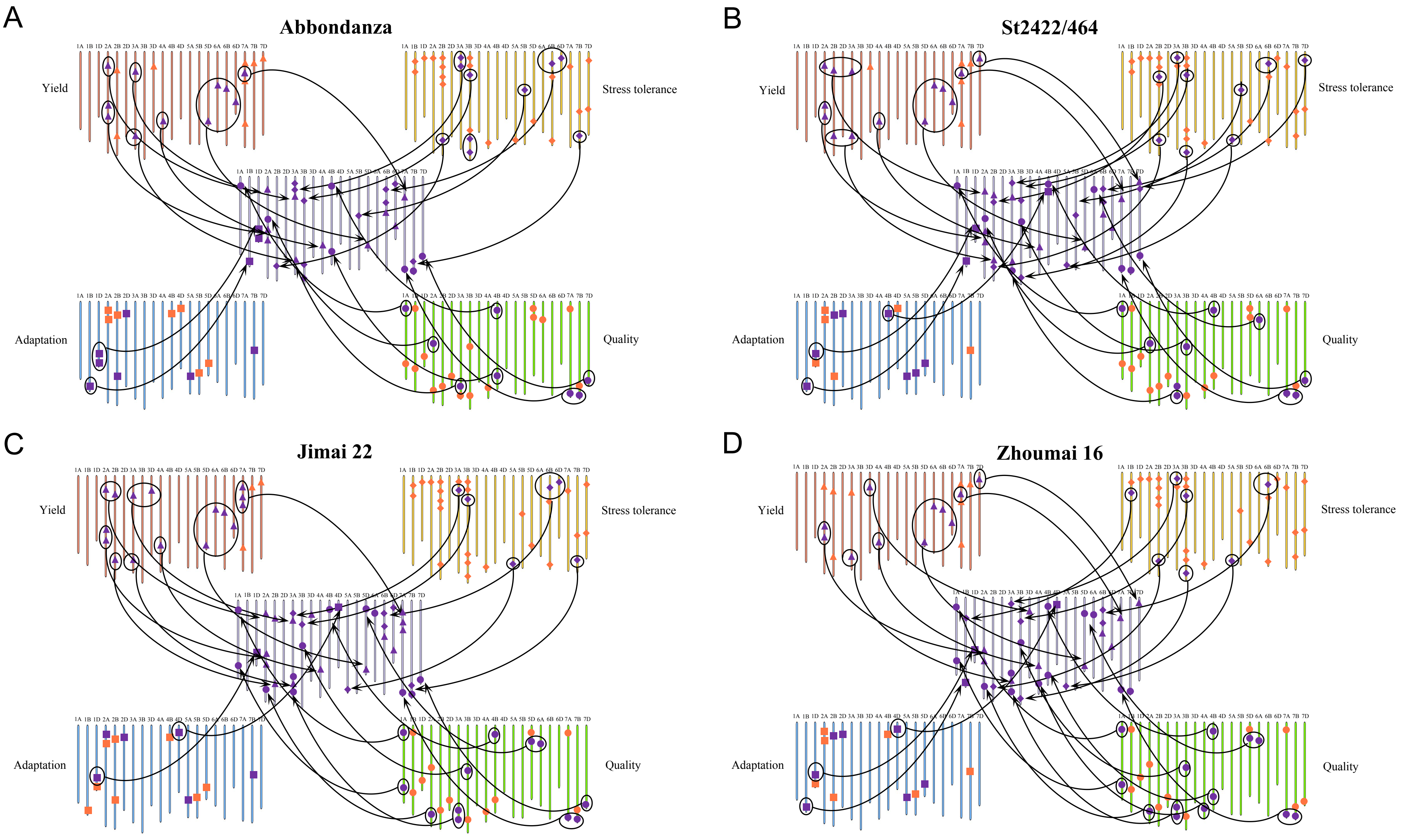

Figure S17. Enrichment of favorable alleles for agronomically important genes in four founder parents. (A) Enrichment of favorable alleles for agronomically important genes in founder parent Abbondanza. Genes for yield, stress tolerance, adaptability, and quality are shown in red, yellow, blue, and green, respectively, distributed among chromosomes. The favorable and alternative alleles of each genes are shown in purple and orange, respectively. Favorable alleles of agronomically important genes are enriched on the middle chromosomes. (B) Enrichment of favorable alleles for agronomically important genes in founder parent St2422/464. (C) Enrichment of favorable alleles for agronomically important genes in founder parent Zhoumai 16. (D) Enrichment of favorable alleles for agronomically important genes in founder parent Jimai 22.
