# Supplementary material for: Conservatively transmitted alleles of key agronomic genes provide insights into the genetic basis of founder parents in bread wheat (Triticum aestivum L.)
Source: BMC Plant Biol. 2023 Feb 18;23:100. doi: 10.1186/s12870-023-04098-x (PMC9938602; doi:10.1186/s12870-023-04098-x)
Supplement: Supplementary file 14 — Additional file 14: Figure S4. Genetic composition and allele frequency of founder parents from different breeding periods. (A) Genetic composition of 87 yield, stress resistance, quality, and adaptability genes in 40 founder parents (FPs) from five different breeding periods. The favorable and alternative alleles are shown in purple and orange, respectively. Heterozygous and missing types are represented by magenta and black, respectively. (B) Allele frequencies of 87 agronomically important genes controlling yield, stress resistance, quality, and adaptability in 40 founder parents from five different breeding periods. The favorable and alternative alleles are shown in purple and orange, respectively. [file 12870_2023_4098_MOESM14_ESM.pdf]

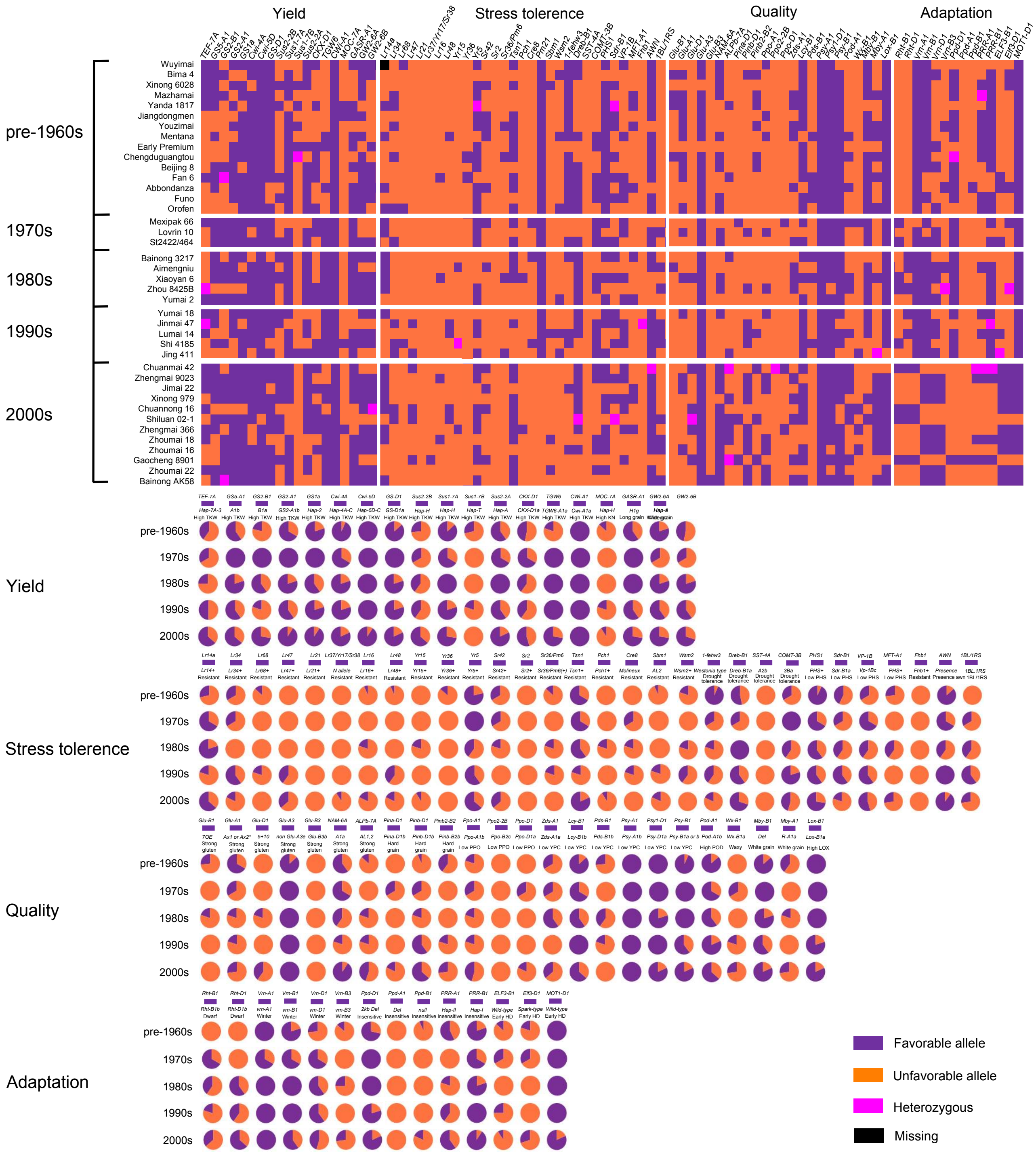


**Figure S4.** Genetic composition and allele frequency of founder parents from different breeding periods. (A) Genetic composition of 87 yield, stress resistance, quality, and adaptability genes in 40 founder parents (FPs) from five different breeding periods. The favorable and alternative alleles are shown in purple and orange, respectively. Heterozygous and missing types are represented by magenta and black, respectively. (B) Allele frequencies of 87 agronomically important genes controlling yield, stress resistance, quality, and adaptability in 40 founder parents from five different breeding periods. The favorable and alternative alleles are shown in purple and orange, respectively.
